# Typical and Atypical Development of Visual Expertise for Print as Indexed by the Visual Word N1 (N170w): A Systematic Review

**DOI:** 10.3389/fnins.2022.898800

**Published:** 2022-06-30

**Authors:** Kathleen Kay Amora, Ariane Tretow, Cara Verwimp, Jurgen Tijms, Paavo H. T. Leppänen, Valéria Csépe

**Affiliations:** ^1^Brain Imaging Centre, Research Centre for Natural Sciences, Budapest, Hungary; ^2^Faculty of Modern Philology and Social Sciences, Multilingualism Doctoral School, University of Pannonia, Veszprém, Hungary; ^3^Department of Psychology, University of Jyväskylä, Jyväskylä, Finland; ^4^Department of Developmental Psychology, University of Amsterdam, Amsterdam, Netherlands; ^5^Rudolf Berlin Center, Amsterdam, Netherlands; ^6^Institute for Hungarian and Applied Linguistics, University of Pannonia, Veszprém, Hungary

**Keywords:** reading development, dyslexia, words, developmental reading disorder (DRD), event-related potentials (ERP), visual expertise, N170, systematic review

## Abstract

The visual word N1 (N170w) is an early brain ERP component that has been found to be a neurophysiological marker for print expertise, which is a prelexical requirement associated with reading development. To date, no other review has assimilated existing research on reading difficulties and atypical development of processes reflected in the N170w response. Hence, this systematic review synthesized results and evaluated neurophysiological and experimental procedures across different studies about visual print expertise in reading development. Literature databases were examined for relevant studies from 1995 to 2020 investigating the N170w response in individuals with or without reading disorders. To capture the development of the N170w related to reading, results were compared between three different age groups: pre-literate children, school-aged children, and young adults. The majority of available N170w studies (*N* = 69) investigated adults (*n* = 31) followed by children (school-aged: *n* = 21; pre-literate: *n* = 4) and adolescents (*n* = 1) while some studies investigated a combination of these age groups (*n* = 12). Most studies were conducted with German-speaking populations (*n* = 17), followed by English (*n* = 15) and Chinese (*n* = 14) speaking participants. The N170w was primarily investigated using a combination of words, pseudowords, and symbols (*n* = 20) and mostly used repetition-detection (*n* = 16) or lexical-decision tasks (*n* = 16). Different studies posed huge variability in selecting electrode sites for analysis; however, most focused on P7, P8, and O1 sites of the international 10–20 system. Most of the studies in adults have found a more negative N170w in controls than poor readers, whereas in children, the results have been mixed. In typical readers, N170w ranged from having a bilateral distribution to a left-hemispheric dominance throughout development, whereas in young, poor readers, the response was mainly right-lateralized and then remained in a bilateral distribution. Moreover, the N170w latency has varied according to age group, with adults having an earlier onset yet with shorter latency than school-aged and pre-literate children. This systematic review provides a comprehensive picture of the development of print expertise as indexed by the N170w across age groups and reading abilities and discusses theoretical and methodological differences and challenges in the field, aiming to guide future research.

**Systematic Review Registration:**

https://www.crd.york.ac.uk/prospero/display_record.php?ID=CRD42021228444.

## Introduction

Reading, which involves successfully and fluently linking letters to sounds, is one of the prerequisites to participate in today's society. Learning to read is commonly shaped through years of exposure to text and formal teaching. Although we are constantly exposed to text, some do not successfully develop fluent reading skills, with the poorest 3–10% of the children being considered to have developmental dyslexia or developmental reading disorder (DRD; Snowling, 2013).

Fast recognition of words is critical for attaining automatized reading in alphabetic orthographies (McCandliss et al., 2003) and is associated with a reorganization of the visual systems that are evolving to process the new word forms efficiently. Event-related potential (ERP) studies have associated the visual N170 component, which peaks around 170 milliseconds after stimulus onset, with the expertise for visual stimuli such as words. The visual word N170 (hereafter referred to as N170w) is a response with a negative deflection commonly largest over occipitotemporal regions, and its lateralization depends on maturation and reading experience (Maurer and McCandliss, 2008). The emergence of N170w is supposedly rooted in the visual word form area (VWFA) within the ventral occipitotemporal cortex (vOTC) of the left hemisphere, which has been known to show sensitivity to visual words throughout literacy (McCandliss et al., 2003; Rossion et al., 2003; Dehaene et al., 2010). Moreover, it has been considered as a neurophysiological marker for print expertise with prelexical sensitivity to letter/character strings (Maurer et al., 2006; Luck, 2012). Higher N1 amplitudes for words than low-level visual control stimuli such as meaningless symbol strings or shapes have been reported across languages (e.g., Dutch: Fraga González et al., 2014, German: Maurer et al., 2006, Portuguese: Araújo et al., 2012).

Several studies have explored the N170 component, which is reported as a category-specific visual expertise marker (Maurer et al., 2008b), and has been studied extensively in face perception studies (e.g., Bentin et al., 1996; Feuerriegel et al., 2015). Other studies have also associated the N170 with sensory processing related to auditory information (Leppänen and Lyytinen, 1997) and referred to the modulation of N170 by attention (Herrmann and Knight, 2001). However, the N170w associated with print tuning has become of particular interest in reading disorder studies in recent years. Aside from the mismatch negativity (MMN) which is commonly used to discuss the role of auditory processing in reading development, the N170w provides a more reading- specific insight related to visual processing for print, which is the primary visual stimuli for reading. Moreover, N170 is reported to possibly predict later reading outcomes as the N170w response is modulated by reading skills (Brem et al., 2013). Furthermore, the N170 has a role in attention, which could be taken into consideration in relation to the visual attention span deficit theory, referring to a higher attention level required in dyslexics for processing of words. Different investigations aiming at characterizing the N170w have identified two different processes; coarse and fine print tuning (e.g., Zhao et al., 2014; Tong et al., 2016a; Kemény et al., 2018). Coarse print tuning, which indicates sublexical processing, entails differential processing of words and non-orthographic symbol strings, whereas fine print tuning usually taps into lexical processes and is required for processing of differences between print and closely matched false font or pseudo-character strings (Maurer et al., 2005; Eberhard-Moscicka et al., 2016). Even though many studies have aspired to shed light on the main visual component with reading development, most of them performed in typical readers or reading disordered individuals have produced contradictory results. These could be due to variability in participant groups, stimuli, and task-specific factors.

Zhao et al. (2014) demonstrated that coarse and even fine-tuning of the N170w can be developed within 1 year of reading instruction. However, N170w print specialization has been found to occur later in children with DRD (Maurer et al., 2007, 2011), suggesting differences in the developmental trajectory of N170w specialization of individuals with DRD compared to their typically developing peers. Longitudinal studies have shown an inverted U-shape development curve of the N170w, with an increased response for orthographic stimuli in beginning readers followed by a slight decrease when readers become fluent (Maurer et al., 2006; Fraga González et al., 2021). However, some studies have shown evidence for a persistent N170w print tuning deficit in individuals with DRD, with no or small differences in the N170w responses to word-like stimuli and matched symbol strings in adults compared to their typically developing peers (Mahé et al., 2012). In addition, for print, it has been found that a bilateral, though somewhat right hemisphere-dominated N170w topography in children changes gradually into left-lateralized topography when reading becomes more automatized. This change occurs shortly after the start of formal reading instruction, contributing to letter-speech sound integration in the form of grapheme-phoneme correspondences (Maurer et al., 2006; Brem et al., 2013). However, for individuals with DRD, the response lateralization showed no consistent pattern: left (e.g., Araújo et al., 2012), bilateral (e.g., Fraga González et al., 2014), or right-lateralized (e.g., van Setten et al., 2019) distributions were reported.

Although numerous studies have demonstrated an atypical N170w response to words in individuals with DRD, the effects regarding amplitude, latency, and lateralization have been inconsistent. Moreover, the variation in experimental designs and setups could pose challenges in interpreting results for interested researchers in the field. Therefore, our systematic review assimilated existing research on typical and atypical development of visual reading processes as reflected by the N170w response. The main objective of this review was to give an overview of the status quo of the N170w literature related to reading development in terms of reading ability (typically vs. atypically developing readers) and age group (from pre-literate age until adulthood). For our secondary objectives, we examined differences in N170w in comparison with other word-like conditions (e.g., pseudowords, nonwords) and the potential impact of various linguistic factors (e.g., language, orthographic depth). In addition, we investigated theoretical and methodological differences applied in the N170w studies to guide future research using this component to investigate typical and atypical reading.

## Materials and Methods

### Protocol and Registration

The protocol for this systematic review was pre-registered and uploaded to https://www.crd.york.ac.uk/prospero/display_record.php?ID=CRD42021228444. All aspects of this review adhered to the Preferred Reporting Items in Systematic Reviews (PRISMA) guidelines (Moher et al., 2009).

### Eligibility Criteria and Study Selection

Studies included in the current review satisfied the following criteria after the full-text review: (1) cross-sectional, longitudinal, and intervention studies on the visual word N1/N170 employing different stimulus conditions, i.e., letter/character strings vs. non-letter/non-character stimuli (case studies, reviews, theses or dissertations, and gray literature were excluded; as well as using single letters only as stimuli was excluded); (2) a sample involving participants with or/and without developmental reading disorders (DRD) (studies that focused only on other neurological/developmental conditions or comorbidities aside from DRD (e.g., ADHD) as well as with impaired hearing or a (severely) visual handicap were excluded); (3) participants that could be categorized into one of the following age groups: pre-literate children (3–6 years old), school-aged/literate children (7–11 years old) and young adults (18–35 years old); and (4) reported findings in an English-language, peer-reviewed journal between 1995 and 2020. The earlier year limitation (1995) was implemented to not have a bias toward earlier works, but also to have a clear limitation that helps in keeping the methodological considerations consistent and comparable (i.e., equipment, sample size), whereas the late year limitation (2020) served as a clear cut-off of the search date when the search terms were applied.

### Systematic Review Procedure

#### Information Sources, Search, Data Collection Process

We searched Web of Science, PubMed (MEDLINE), PsychINFO, PubPsych, ProQuest, Scopus, PsycNET, and Cochrane for studies using the following search strings: (N1 OR N170) AND (EEG OR ERP OR event-related potential^*^) AND (visual OR word OR print) AND (expertise OR read^*^ OR develop^*^) AND [read^*^ AND (disorder^*^ OR disab^*^ OR dyslexi^*^ OR difficult^*^ OR problem^*^ OR develop^*^)] AND (participant^*^ OR child^*^ OR adult^*^).

Final searches were conducted on the 11th and 19th of January 2021. The articles underwent four rounds of screening: removal of duplicates, abstract screening, full-text reading, and data extraction. Removal of duplicates, title, and abstract screening were performed using the Rayyan software for systematic reviews (Ouzzani et al., 2016). The evaluation process was conducted by three independent raters, with title and abstract screening being performed fully blinded.

#### Risk of Bias in Individual Studies

Included studies underwent a risk-of-bias assessment using the Newcastle—Ottawa Scale (NOS) adapted to cross-sectional studies (Modesti et al., 2016). Each rater judged every study based on seven quality items categorized into three sections: the study group selection (representativeness of the sample, sample size, non-respondents, measurement tool for assessment of reading skill), the comparability of the groups; and the outcome (assessment and statistics). Each rater awarded a star per item if the study fits the criteria. Obtained NOS scores (*M* = 7, *SD* = 2) were reported in Supplementary Table S1. Interrater reliability was assessed through percentage agreement of rater1, rater2, and rater3 of the NOS. For this, 10% of the reviewed studies (*n* = 7) were randomly selected and reassessed by the second and third rater. Interrater reliability between each rater pair was 71.24% (R1/R2, R1/R3, R3/R2).

#### Data Items

The following data were extracted from all selected papers: participant information (e.g., sample size, participant age, reading ability groups), EEG parameters (e.g., pre-processing steps and region/scalp areas of interest as defined by electrode set used in the analyses), stimuli and task characteristics (e.g., language, experimental design), and ERP results (i.e., amplitude, lateralization, latency). We based our ERP summary on the statistical results and the graphical representations present in the text. The full details of the extracted data can be found in Supplementary Table S2.

#### Synthesis of Results

We employed a narrative synthesis to compile the results regarding N170w, amplitude, latency, and lateralization of the selected studies and provided summary tables that included essential extracted features of the study (e.g., participants, age, task, results). In extracting the results for individual studies, we excluded ERP results using other forms of analyses (e.g., topographic analysis of variance, LORETA). The original scope of means and effect sizes extraction of the selected papers had to be reviewed due to the lack of reported means and effect sizes in the papers included in this review. For evaluation of lateralization and amplitude, variables were introduced, which enabled comparison across papers despite the missing mean and effect sizes (i.e., C>DRD, referring to the amplitude of control subjects being enhanced compared to subjects with reading difficulties). Effects of intervention studies on N170w were not assessed; thus, the pre-intervention EEG data only was used for data extraction on N170w for those studies involving training.

## Results

### Study Selection

**Figure 1:**
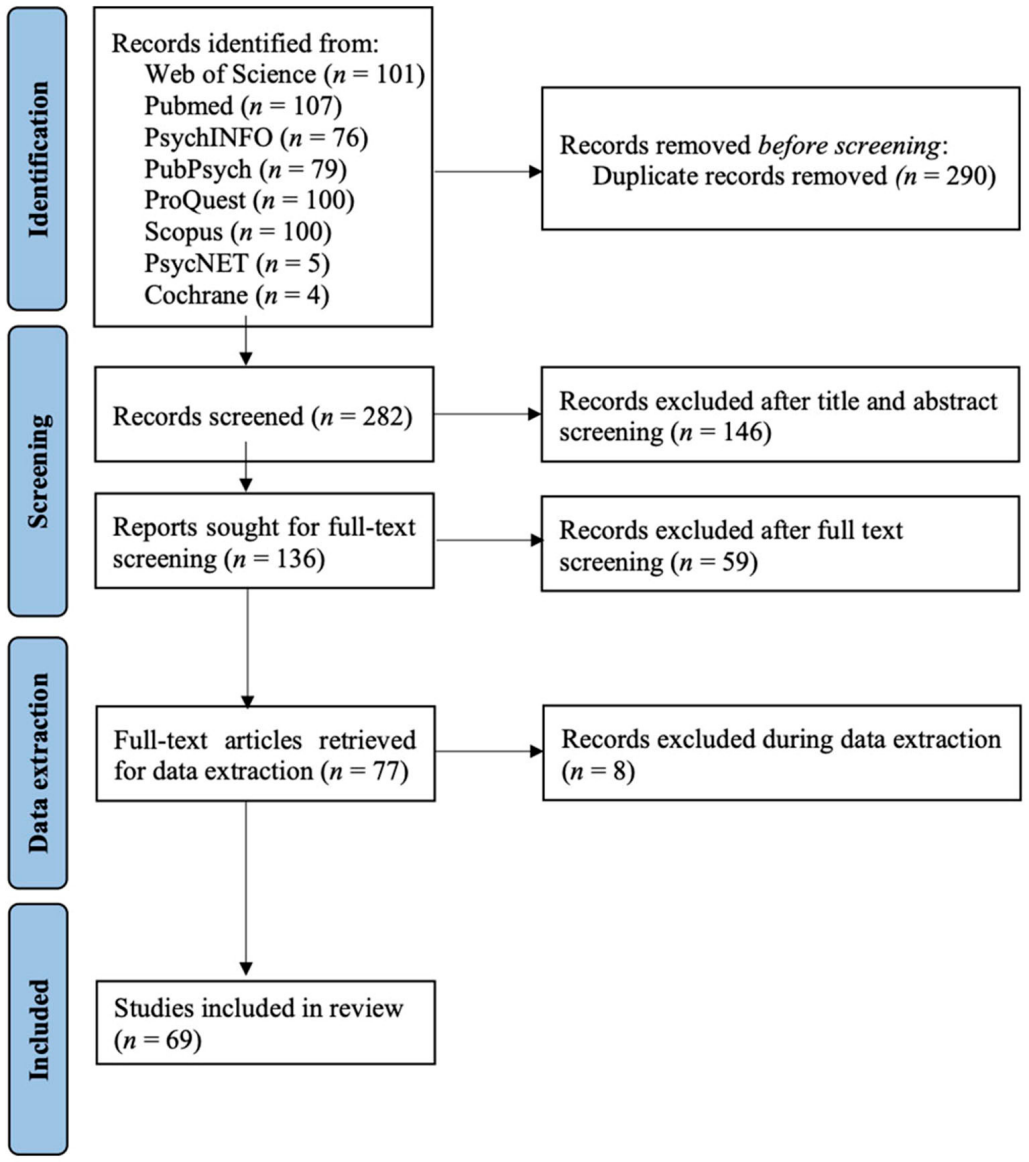
PRISMA flow diagram of the article search, screening, and selection methods. Design adapted from Page et al. (2021).

The initial database search identified 572 articles. Out of 282 non-duplicates, 146 articles were excluded after title and abstract screening using the Rayyan software for systematic reviews (Ouzzani et al., 2016), leaving a number of 136 articles in the full-text screening. All articles were reviewed by authors K. K. A., A. T., and C. V. with a two out of the three-majority decision for inclusion. Twelve conflicting articles were additionally reviewed by the remaining co-authors, leading to the inclusion of two out of twelve articles. After applying the inclusion and exclusion criteria, 59 articles were excluded during full-text screening and eight articles during data extraction, resulting in 69 articles included in the review. A flowchart of this selection process is displayed in Figure 1.

**Figure 2:**
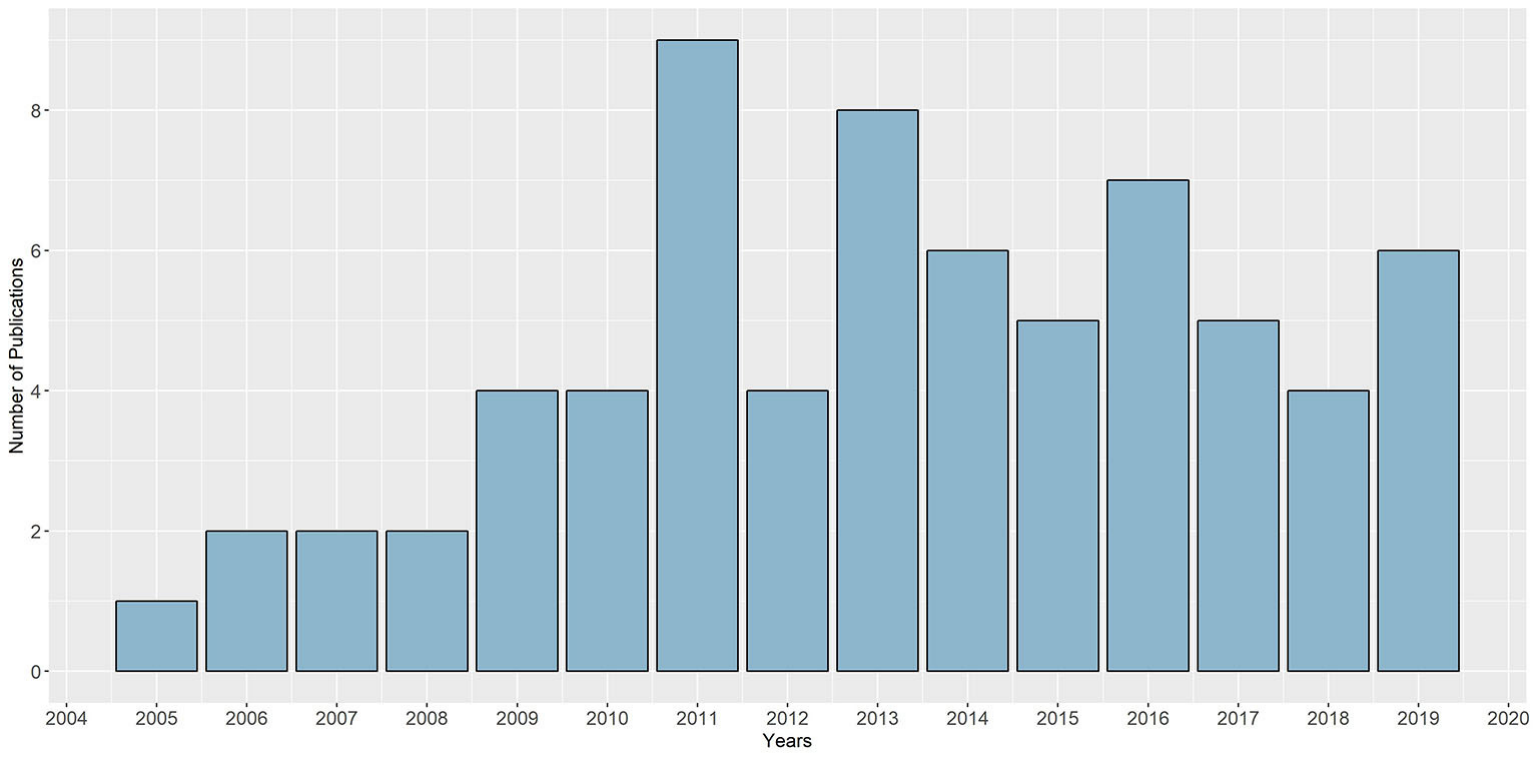
Distribution of included studies across publication years.

A normal distribution across publication years is significantly noticeable among the included articles (see Figure 2). Dense publication years were 2011 (*n* = 9) and 2013 (*n* = 8). Specific characteristics of each of the studies can be found in Supplementary Table S2.

### Methodological Characteristics

#### Participants

Of the 69 studies included in this systematic review, eight examined the N170w in pre-literate children, 31 in school-aged children, three in adolescents, and 41 in young adults aged between 18 and 35. The total number exceeds 69 studies, as 12 of these included more than one age group. The results of the three studies that examined the N170w in adolescents, are combined with the young adult group, as the mean age of the adolescents (*M*_*age*_ = 17.24 years) was close to our lower edge of the young adults age range, and the reported results in terms of amplitude and lateralization were comparable to the results in adults. A substantial number of studies only included typical readers (*n* = 42), whereas 27 studies compared controls with people with dyslexia only (*n* = 23) and/or otherwise defined sample (i.e., poor readers or spellers, illiterate or at-risk individuals; *n* = 8). The number of participants included in each of the studies demonstrated a wide range from 11 to 72. The exact values for each of the reviewed studies together with participant, age, and gender distribution can be obtained *via* Supplementary Table S2. Criteria to consider participants as reading impaired or control varied widely across studies. Participants were considered reading impaired based on either a formal dyslexia diagnosis or the evaluation of reading scores below the 25th, 20th, 16th, and 10th percentile; or 1.5 or 1 standard deviation below the average. On the other hand, typical readers had percentile scores above 10 to >25 in reading tests. These lead to discrepancies across studies as DRD and TD readers overlap across studies reporting criteria (*n* = 23).

#### Language, Stimuli, and Procedure

Most of the studies were conducted in German-speaking populations (*n* = 17), followed by English (*n* = 15), and Chinese (*n* = 14). A minority of five studies investigated a second language. Paradigm types varied between repetition-detection-task (*n* = 16), lexical decision task (*n* = 16), N-back task (*n* = 6), and other paradigms (*n* = 31). All 69 studies used words as a condition, and either had it as the only condition (*n* = 7) or compared words to pseudowords (*n* = 10), pseudo-homophones (*n* = 2) or non-words (*n* = 1). Other comparisons were made to symbols (*n* = 13), faces (*n* = 5) or pictures (*n* = 2). Thirty studies used more than two conditions, mainly comprising words, pseudowords and symbols (*n* = 20). For a detailed overview of all stimuli per study we refer to Supplementary Table S2.

Words presented had an average character length of *M* = 6.62 (*SD* = 2.39, 3–13) letters or strokes. When reported, the word frequency of words commonly ranged in high (*n* = 23) or low to high (*n* = 10) frequency values.

Stimuli duration of words across studies varied between 100 and 5,250 ms, which differed across participants age groups: adults *M* = 489.22, *SD* = 317.49; school-aged children *M* = 845.77, *SD* = 724.62; pre-literate children *M* = 1,125, *SD* = 1683.96. Paradigm difference in stimulus duration was visible for the bigger clusters of detection tasks (*M* = 550.31 ms, *SD* = 460.19) and lexical decision tasks (*M* = 784.38 ms, *SD* = 903.09). The explicit word/symbol processing task (5,250 ms) and dual valence task (100 ms) were the most deviating paradigms. The number of presented trials was another dividing factor, ranging from 40 to 576 trials for the word conditions (Brem et al., 2013; Collins et al., 2017). Distance to screen for the word presentation ranged from 50 cm to 145 cm (*M* = 81.59 cm, *SD* = 23.82 cm) across studies. Interstimulus intervals (ISI) were composed of different components (e.g., fixation cross and blank screen) across studies. Common feedback, response screens, and blink screens were among the reported procedures for the composition of trials (see Supplementary Table S2).

#### EEG Analysis

The presented studies (*N* = 69) had a significant difference in the number of EEG channels recorded (19–128, *M*_*adult*_ = 64.57, *SD*_*adult*_ = 38.67; *M*_*litchild*_ = 68.23, *SD*_*litchild*_ = 41.27; *M*_*prelitchild*_ = 44.13, *SD*_*prelitchild*_ = 13.29). Across all studies, most common electrode setups were 64 (*n* = 17) and 128 (*n* = 16) electrodes, with one additional study having both setups. Electrodes were reported as Ag/AgCl (*n* = 53), TiN (*n* = 7), implemented in caps of various manufacturers (see Supplementary Table S2). For EGI systems, the common impedance threshold laid at 50 kΩ; for other systems, it varied between the 5–20 kΩ threshold, with a high distribution across systems and studies in general (5–100, *M* = 22.40, *SD* = 22.31). EEG data were recorded at various sampling rates, ranging from 200 to 2,048 Hz. Most studies did not report on downsampling procedures (*n* = 55); if reported, we recorded values between 256 and 500 Hz. While the reference electrodes used varied across studies (e.g., mastoid, nose tip, Cz, and Biosemi CMS/DRL), re-referencing to the average was a common practice (*n* = 50) as preprocessing step. Other re-referencing methods were reported as Cz, average of mastoids, and multi-electrode referencing (Simon et al., 2007: using 20 out of 32 electrodes, F7, F3, C3, T3, CP3, TP7, T5, P3, F8, F4, C4, T4, CP4, TP8, T6, P4, Fz, Cz, Cpz, and Pz). During recording, common online filtering ranged between 0.1 and 100 Hz. Further, low- (20–48 Hz) and high-pass (0.01–1 Hz) filters were applied. Common baseline windows ranged between 50 and 500 ms pre-stimulus, whereas the most used time frames for baseline were at 100 ms (*n* = 28) and 200 ms (*n* = 18) pre-stimulus onset. A difference between the applied baseline windows was visible between pre-literate and other age groups (*M*_*prelit*_= −112.5, *SD* = 13.36, *M*_*other*_ = −154.68, *SD* = 81.50), possibly related to the small number of papers *(n* = 8) targeting pre-literate population. Independent Component Analysis (ICA) for ocular artifacts and automated artifact rejection with threshold (between ±80 and 125 μV) was commonly reported; if manual rejection was performed, it was commonly performed in combination with another approach. The number of trials included after artifact rejection was sparsely reported.

Regarding the further analysis, the epochs around the target word varied across studies, ranging in the length of the epochs from 250 ms to 2 s, *M* = −158.98 ms (−500–0) to *M* = 860.03 ms (250–1,550). The timeframe in which the peak of N170w was obtained in studies regarding the three age groups differed significantly between adult and child groups (pre-literate children: 175–238.5, *M* = 216.56, *SD* = 20.53; school-aged children: 175–238.5, *M* = 215.25, *SD* = 16.43; adults: 150–270, *M* = 183.01, *SD* = 22.29). These studies have mostly used either global field power (GFP) analyses (*n* = 23), visual peak detection (*n* = 14), or literature reviewing (*n* = 7) for selection of the N170w time window. The regions of interest (ROI) examined for N170w have varied across studies, though most studies focused on P7 (*n* = 47), P8 (*n* = 38), and O1 (*n* = 36). N170w amplitudes were obtained using the mean amplitude of the identified ERP time window (*n* = 37) or maximum peak amplitude within the ERP time window (*n* = 21). A lack of reported mean values of the N170w amplitudes to words was observed in most studies, with reliance on the presentation of the mean amplitudes in graphs and ERP waveforms. This form of presentation led to the analysis of N170w amplitude being limited to a qualitative approach of the presented graphs, as also presented statistical results did not include word condition only results.

#### Statistical Analysis

Forty of the reviewed studies obtained their statistical results by applying analysis of variance (ANOVA). Multivariate analysis of variance (MANOVA) was performed in six studies. Greenhouse-Geisser, Tukey HSD, or Bonferroni corrections were mentioned to be applied by nine studies. Linear models were in the minority, with three applications across studies. Meanwhile, a *t*-test as a lone standing evaluation of N170w specific values was reported by two studies. Between-subject factors across studies were group, age, gender, reading level, hearing level, and others. Within-subject factors mainly consisted of condition and stimuli features and hemispheres/electrode site. Commonly, the study design and statistical computations were not designed to be investigating the N170 response to words alone.

### Results of Individual Studies

The full details of the extracted results can be found in Supplementary Table S3.

#### N170w in Typically Developing vs. Developmental Reading Disorder/Poor Readers

Results are reviewed by age group relative to the number of studies that compared different reading ability groups (typically developing: TD, and atypically developing such as developmental reading disorder/poor readers/low reading ability: DRD/PR). Some studies that used the term “Developmental Dyslexia/Dyslexia” are referred to as DRD in this paper. Amplitude, latency, and lateralization comparisons for each age group are displayed in Tables 1, 2.

**Table 1:** N170w Amplitude and Latency results in comparing TD and DRD/PR by age group.

**Studies**	**Amplitude (*N* = 29)**				**Latency (*N* = 7)**			
	**C > DRD/PR**	**C < DRD/PR**	**C = DRD/PR**	**Total**	**C > DRD/PR**	**C < DRD/PR**	**C = DRD/PR**	**Total**
Pre-literate	1	0	2	3	–	–	–	0
School-aged	5	5	4	14	1	–	4	5
Young adults	11	1 (right)^*^	1 (left)^*^	12	–	2	–	2

*Counts in each column refer to the number of studies reporting that result*.

* *Different sub-groupings in one study (Dujardin et al., 2011)*.

**Table 2:** N170w Lateralization results in comparing TD and DRD/PR by age group.

**Studies**	**Lateralization (*N* = 20)**				
	**C = left** **DRD/PR = equal**	**C = right** **DRD/PR = equal**	**C = left** **DRD/PR = right**	**C = equal** **DRD/PR = right**	**No difference**
Pre-literate (*n* = 2)	1	0	0	1	0
School-aged (*n* = 11)	1	3	0	0	4 (bilateral), 2 (left), 1 (right)
Young adults (*n* = 7)	6^*^	0	1^*^	0	2 (left)^*^

*Counts in each column refer to the number of studies reporting that result*.

* *Different sub-groupings in one study (Dujardin et al., 2011: C, left, DRD1, left at trend level, DRD2, bilateral; Mahé et al., 2013: C, left; PR, bilateral, DRD, right at trend level)*.

##### Amplitude

Forty studies investigated the N170w amplitude in TD and DRD/PR. A total of 29 studies compared the N170w amplitudes between TD and DRD/PR individuals in pre-literate children (*n* = *3*), school-aged children (*n* = *14*), or young adults (*n* = *12*).

In pre-literate children, only three studies investigated the N170w between TD and at risk of DRD/PR. Studies revealed contradictory results, wherein one found larger N170w amplitudes in controls (Li et al., 2013), and two found no amplitude differences between TD and at-risk of DRD/PR groups (Maurer et al., 2007; Brem et al., 2013).

Thirty-one studies explored the N170w in school-aged children, of which 14 compared TD with DRD/PR. Five studies showed a larger N170w amplitude for DRD/PR as compared to controls (Brem et al., 2013; Fraga González et al., 2014, 2016b; Zhao et al., 2014; van Setten et al., 2019), five showed a larger N170w amplitude for controls than DRD/PR (Maurer et al., 2007, 2011; Jucla et al., 2010; Kast et al., 2010; Bakos et al., 2018), and four showed no difference (Araújo et al., 2012; Hasko et al., 2013; Kemény et al., 2018; Pleisch et al., 2019). One specific study further divided the TD and DRD children into young (*M*_*age*_ = 8.3) and old (*M*_*age*_ = 11.4) sub-groups and found that in younger groups, TD exhibited a more negative N170w than DRD/PR, whereas the opposite pattern was found for older children (Maurer et al., 2011).

Forty-two studies investigated the N170w amplitude in young adults, of which 12 compared TD and DRD/PR groups. Eleven studies showed that controls exhibited a larger, thus more negative, N170w than DRD/PR subgroups (Savill and Thierry, 2011a,b; Korinth et al., 2012; Mahé et al., 2012, 2013; Waldie et al., 2012; González-Garrido et al., 2014; Korinth and Breznitz, 2014; Araújo et al., 2015; van Setten et al., 2016; Collins et al., 2017). One specific study examined two subgroups of people with DRD based on the inspection of the ERPs; one that exhibited an N170 but no N320 and one with the two waves fused together (Dujardin et al., 2011). The authors found no difference on N170w amplitudes between TD and the first subgroup of DRD over the left hemisphere, but the latter showed more negativity than the former on the right hemisphere electrodes.

##### Latency

Only 20 out of 69 selected studies explored the latency of the N170w. Thirteen of these provided specific mean latency values. Reported latency results were mainly from the studies comparing different groups (TD vs. DRD/PR or age). Some studies also analyzed the N170w latency values regarding hemispheric distribution (left vs. right) within participant groups.

Eight studies compared the mean N170w latencies of TD and DRD/PR groups. No such studies were conducted in pre-literate TD and at-risk of DRD/PR children. In school-aged children, four studies showed similar mean latencies for both TD and DRD/PR groups (Kast et al., 2010; Maurer et al., 2011; Hasko et al., 2013; Zhao et al., 2014), whereas one study reported that controls had longer mean latencies than DRD (van Setten et al., 2019). In young adults, two studies reported longer mean latencies for DRD than controls (Savill and Thierry, 2011a; Waldie et al., 2012).

van Setten et al. (2016, 2019) were interested in the assumed interaction of mean N170w latency and hemispheric distribution in TD and DRD groups and found a significantly longer mean N170w latency in the right hemisphere compared to the left in both TD and DD/PR groups in young adults and school-aged children.

##### Lateralization

Out of all the selected 69 studies, 61 investigated the lateralization of the N170w. However, only 20 compared the lateralization between typically developing and reading impaired participants.

In pre-literate children, two studies compared the N170w lateralization between TD and at-risk of PR. Li et al. (2013) reported a left-lateralized N170w for controls, but bilateral activity in at-risk of PR. In contrast, Brem et al. (2013) found bilateral activity for pre-literate controls and a right-dominated N170w, although only at a trend level for pre-literate at-risk of PRs.

Twenty-seven studies investigated the lateralization of the N170w in school-aged children, of which 11 compared TD and DRD/PR groups. Out of the 11 studies, seven studies showed no difference in hemispheric dominance of the N170w between TD and DRD/PR children: four studies reported bilateral activation (Jucla et al., 2010; Hasko et al., 2013; Kemény et al., 2018; Pleisch et al., 2019), two reported left (Maurer et al., 2011; Araújo et al., 2012) and one reported right activation preponderance (van Setten et al., 2019) in both groups. The remaining four studies reported either left (Kast et al., 2010) or right-lateralization (Fraga González et al., 2014, 2016b; Zhao et al., 2014) for controls only, but found bilateral activity in DRD/PR children. To conclude, lateralization in DRD/PR school-aged children was mainly reported to be bilateral (*n* = 8) and control school-aged children appeared to show left, right and bilateral dominance (*n* = 11, *n* = 7, *n* = 12).

Thirty-six studies on N170w lateralization were found in young adults. Seven of these compared TD and DRD/PR groups, of which six studies showed left lateralization of the N170w for the controls and a bilateral activation for DRD/PR groups (Dujardin et al., 2011; Mahé et al., 2012, 2013; González-Garrido et al., 2014; Araújo et al., 2015; Collins et al., 2017). Aside from bilateral activation, the other DRD subtype in Dujardin and colleagues' (2011) study showed left lateralization of the N170w, though at trend level only. Moreover, one study showed left-lateralization for both TD and DRD (van Setten et al., 2016), and another study found left-lateralization for controls but investigated poor readers and adults with DRD separately and found that the former exhibited a bilateral activation of the N170w whereas the latter showed a right-lateralized N170w at trend level (Mahé et al., 2013). These results indicate a clear left-hemispheric distribution for typical reading adults (*n* = 33), with more bilateral distribution occurrences in reading impaired adults (*n* = 6).

#### N170w From Pre-literate Age to Adulthood

Eighteen studies gave additional insights on the development of N170w amplitude by including different age groups using a cross-sectional or longitudinal design. These studies mainly evaluated control subjects (Maurer et al., 2005, 2006, 2007, 2011; Brem et al., 2006, 2009, 2013; Spironelli and Angrilli, 2009; Van Strien et al., 2009; Cao and Zhang, 2011; Cao et al., 2011; Dundas et al., 2014; Coch and Meade, 2016; Eberhard-Moscicka et al., 2016; Tong et al., 2016a; Curzietti et al., 2017; van Setten et al., 2019; Zhao et al., 2019).

##### Amplitude

Only two studies compared the N170w across pre-literate age, school-aged, and adulthood in typically developing individuals (Maurer et al., 2006; Eberhard-Moscicka et al., 2016). Eberhard-Moscicka et al. (2016) investigated the development of the N170w in the context of foreign language learning (English). However, the results in this review only included N170w response to the stimuli in the native language, German. Both authors found that N170w amplitudes consistently decreased in adults. However, two studies showed a reversed effect in the children groups, wherein Eberhard-Moscicka et al. (2016) showed a decrease of N170w amplitude from pre-literate to school-children, and Maurer et al. (2006) found the opposite: school-aged children produced a larger N170w amplitude compared to pre-literate children. Two other studies included TD school-aged children, adolescents (*M*_*age*_ = 16.2 years), and adults: the adolescents exhibited a larger N170w compared to adults (Brem et al., 2006, 2009) but smaller when compared to school-aged children (Brem et al., 2009).

Eleven studies compared two TD age groups. Maurer et al. (2007) found that pre-literate children exhibited smaller N170w amplitudes than school-aged children. Five studies compared TD school-aged children and adults (Spironelli and Angrilli, 2009; Cao and Zhang, 2011; Cao et al., 2011; Coch and Meade, 2016; van Setten et al., 2019), whereas one study compared pre-literate children and TD adults (Maurer et al., 2005). All found similar results, i.e., larger N170w amplitudes in children compared to adults.

In addition, four papers compared young (*M*_*age*_= 8) and old school-aged (*M*_*age*_ = 11) children and collectively corroborated the finding of Maurer et al. (2011), i.e., larger N170w amplitudes in younger children compared to the older group (Van Strien et al., 2009; Cao et al., 2011; Tong et al., 2016a; Zhao et al., 2019). One study divided the adults into young (20–30 years old) and old (>40 years old) groups, wherein the latter exhibited a larger N170w than the former (Curzietti et al., 2017). Lastly, one study compared gender differences, with boys showing larger N170w amplitude than girls (Spironelli et al., 2010).

##### Latency

Six studies compared the mean latencies of two or three age groups. Five of these showed that the N170w peaked earlier in adults than in pre-literate children (Maurer et al., 2005), school-aged children (Brem et al., 2009; Cao and Zhang, 2011; Cao et al., 2011), and adolescents (Brem et al., 2006). Only one study showed similar mean latencies in school-aged children and adults (Coch and Meade, 2016).

Five studies investigated the interaction of mean latency and hemispheric distribution of the N170w. Three studies were conducted on pre-literate children and revealed opposite results. (Zhao et al., 2015) reported in their training study that the N170w occurred later over the right than the left hemisphere for the visual learning group (visual identification of characters); however, they saw a reversed pattern in the writing condition group (manual tracing and copying of characters) at the pre-test phase before training. The same research group (Zhao et al., 2018) found, according to their earlier finding, that the N170w latency was only slightly delayed in the right hemisphere compared to the left (Zhao et al., 2018), and another study did not find any latency differences between the hemispheres (Maurer et al., 2005). To examine whether the reported latencies across studies differed significantly between the hemispheres, we conducted a two-tailed t-test, which did not reveal significant differences across the three studies presented for pre-literate children (*M*_*left*_ = 215.5, *SD*_*left*_ = 7.5; *M*_*right*_ = 217.2, *SD*_*right*_ = 5.1). Two studies divided their school-aged sample into a young and old subgroup (Maurer et al., 2011; Tong et al., 2016a). Maurer et al. (2011) found a longer latency for the younger children compared to the older ones, whereas Tong et al. (2016a) reported the opposite pattern. However, school-aged children generally showed nearly no differences in the mean N170w latencies between the left and right hemispheres (M_*left*_ = 214.7 ms, M_*right*_ = 215.5 ms). For young adults, controversial latency values have been reported, with a longer mean latency of the N170w over the right hemisphere than the left in one study (van Setten et al., 2016), and the opposite was observed in another one (Xue et al., 2019). Compared to pre-literate and school-aged children, the N170w occurred earlier in adults (*M*_*left*_ = 161.2 ms, *M*_*right*_ = 161.9 ms).

##### Lateralization

Nine studies compared the N170w lateralization across different age groups. Two studies found that pre-literate and school-aged children exhibited bilateral N170w, but this N170w became left-lateralized in adulthood (Maurer et al., 2006, 2007). However, one finding showed that pre-literate children exhibited right-lateralized N170w, which became left-lateralized in adulthood (Maurer et al., 2005). Other findings either showed a left-lateralized N170w (Cao and Zhang, 2011; Cao et al., 2011; Dundas et al., 2014), a bilateral distribution (Mercure et al., 2009), or a right-lateralized N170w (Spironelli and Angrilli, 2009) in childhood that became left-lateralized in adulthood (Mercure et al., 2009; Spironelli and Angrilli, 2009; Cao and Zhang, 2011; Dundas et al., 2014). Lastly, one study investigated school-aged children, adolescents, and adults and found no differences in the lateralization across the age groups measured (Brem et al., 2009).

Many studies investigated lateralization of the N170w in one age group only. For pre-literate children, bilateral activity was reported in two studies of the same research group (Zhou et al., 2009; Zhao et al., 2015). Nine studies investigated the N170w lateralization in school-aged children. Five reported left-lateralization of the N170w (Van Strien et al., 2009; Cao et al., 2011; Su et al., 2015; Sacchi and Laszlo, 2016; Bakos et al., 2018), and two reported no difference between the responses recorded over the two hemispheres (Eberhard-Moscicka et al., 2015; Tong et al., 2016a). Another study on TD school-aged children compared two types of orthographic scripts, alphabetic and logographic, and found a right-lateralized N170w for the former and a bilateral distribution for the latter (Tong et al., 2016b). Lastly, one study compared gender differences in TD school-aged children, wherein girls exhibited right dominance and boys showed bilateral activity (Spironelli et al., 2010). Twenty-two studies investigated TD young adults only, and the vast majority (*n* = 20) of these studies showed significant left-lateralization (Brem et al., 2006; Simon et al., 2007; Maurer et al., 2008a,b; Lin et al., 2011; Mercure et al., 2011; Yum et al., 2011; Korinth et al., 2012; Taha and Khateb, 2013; Taha et al., 2013; Okumura et al., 2015; Curzietti et al., 2017; Emmorey et al., 2017; Uno et al., 2017; Yang et al., 2017; Wei et al., 2018; Davis et al., 2019; Faísca et al., 2019; Xue et al., 2019) or at trend level (Maurer et al., 2010). In contrast, two studies reported either bilateral (Okumura et al., 2014) or right lateralization (Cao et al., 2013). An overviewof the lateralization results is displayed in Table 3.

**Table 3:** N170w Lateralization results in comparing age groups in studies that only included a TD group.

**Studies**	**Lateralization (*N* = 33)**		
	**Left > Right**	**Left < Right**	**Left = Right**
Pre-literate (*n* = 2)	0	0	2
School-aged (*n* = 9)	5	2^*^,^**^	4^*^,^**^
Young Adults (*n* = 22)	20	1	1

*Counts in each column refer to the number of studies reporting that result*.

* *Different scripts: Tong et al., 2016b- alphabetic: right-lateralized N170w and logographic: bilateral distribution*.

** *Different gender: Spironelli et al., 2010 − girls: right-lateralized, boys: bilateral distribution*.

#### N170w vs. Word-Like Conditions

To investigate the N170 response related to early lexical effects, we included studies that, aside from words, used word-like stimuli. These word-like conditions consisted of pseudowords (PW, resembles the orthographic and phonological structure of a real word, thus pronounceable), pseudo-homophones (PH, sounds like a real word but incorrectly spelled), and non-words (NW, orthographically or phonologically illegal letter strings that are not pronounceable, excluding symbols and false fonts).

##### Amplitude

Within-subject manipulations of word-like stimuli such as comparing words vs. pseudowords, pseudo-homophones, or non-words were examined in 21 studies, mostly in typically developing individuals, and eight of these compared TD and DRD/PR groups. Three studies compared two different age groups, i.e., school-aged children and adults. Furthermore, one study investigated these word and word-like conditions in pre-literate children only, seven studies involved exclusively school-aged children and ten studies included only young adults.

Mean amplitudes between word and word-like comparisons in a Chinese study on pre-literate children revealed a higher amplitude for line and character conditions compared to the stroke (re-arrangement of stroke combinations in a radical) and radical (non-character stimulus due to illegal position of radicals) conditions in the left hemisphere (Zhao et al., 2018). The left hemisphere showed an overall, more robust N170 response. In school-aged children, results showed either a more negative N170 response for pseudowords in TD compared to DRD children (Kast et al., 2010) or no difference in TD vs. DRD (Hasko et al., 2013), particularly in the right hemisphere (Zhao et al., 2014). Zhao et al. (2014) showed that the processing of words, pseudowords, and non-words in the left hemisphere varied across TD and PR children. More specifically, the N170 responses to words were more negative than non-words in TD, whereas no difference was found for PR children. In addition, the responses elicited by words were more negative than to pseudowords in PR children, whereas no difference could be found in TD. Moreover, the responses on pseudoword vs. non-word comparisons on the left hemisphere showed a trend level in TD children, with pseudowords showing a more negative N170 than non-words, but no difference between pseudowords and non-words was found for PR children.

For school-aged children, most studies revealed no differences in the N170 response between word and pseudoword conditions (Eberhard-Moscicka et al., 2015; Tong et al., 2016a; Zhao et al., 2019). However, Zhao et al. (2019) corroborated these findings for the right hemisphere only for their older subgroup in the same study, but they found a more negative N170 amplitude for pseudowords compared to words in the left hemisphere. In comparing words and non-words, one study showed no difference (Pleisch et al., 2019), and another study found a more negative N170 response for words than non-words in the left hemisphere (Tong et al., 2016a).

In six studies on TD young adults, five studies found no difference between words, pseudowords, pseudo-characters, or non-words (Simon et al., 2007; Lin et al., 2011; Okumura et al., 2014; Wei et al., 2018) and one study showed a larger N170 in pseudo-homophones than words (Taha and Khateb, 2013). These results seemed to be moderated by the task design, as these results were confirmed in an explicit task but showed different results in an implicit task, i.e., a larger N170 for words than pseudowords (Faísca et al., 2019). In comparing TD and DRD/PR young adult groups, results showed diversity, wherein (a) words showed less negative N170 than pseudo-homophones and pseudowords in both TD and DRD adults (Araújo et al., 2015); (b) found no difference between words and non-words in the TD group but a more negative N170 to non-words than to words in DRD (Waldie et al., 2012); (c) recorded more negative N170 to words than to non-words and pseudowords in the TD group, but found no difference in the N170 between words and pseudowords in the DRD group over the left hemisphere (Mahé et al., 2012, 2013); or (d) more negative N170 was recorded over the left hemisphere to pseudowords in the TD group, with an opposite result for the DRD group (Dujardin et al., 2011).

Lastly, while Cao and Zhang (2011) found no difference between word and word-like conditions between school-aged children and adults, two studies reported more negative N170w than by pseudowords in TD adults, with no difference for school-aged children (Coch and Meade, 2016; Eberhard-Moscicka et al., 2016). Moreover, one study comparing the N170 to pseudowords and non-words showed no difference in school-aged and young adult groups (Coch and Meade, 2016).

##### Latency

Only six studies investigated the latency differences comparing the N170 recorded in word and word-like conditions. Coch and Meade (2016) compared the N170 latencies of pseudowords and non-words in school-aged children and adults and found longer latency of the N170 responses in children in both conditions compared to adults. In pre-literate children, the N170 for Chinese characters occurred earlier over the left hemisphere than for radical and stroke combinations, whereas in the right hemisphere, the N170 for radical combinations occurred first, followed by that for stroke and character combinations (Zhao et al., 2018). In school-aged children, Zhao et al. (2014) compared TD and PR and found that for the TD group, non-words elicited longer latency responses in both left and right hemispheres than in the PR group. However, the N170 latency to pseudowords and words differed based on the hemisphere, a later response to pseudowords than words over the left hemisphere, and a faster appearing response over the right in both TD and PR groups.

Meanwhile, pseudowords showed the longest latency for the PR group, followed by words and non-words in the left hemisphere, whereas in the right hemisphere, the PR group showed similar results with the TD group, i.e., shortest latency for pseudowords followed by words and non-words. Hasko et al. (2013) found that the N170 for pseudo-homophones and pseudowords had a shorter latency than for words, and this result did not differ between the TD and DRD children. However, Bakos et al. (2018) reported the opposite; the N170 for words exhibited a shorter latency than for pseudo-homophones, but again no differences were found between the TD and DRD groups. Coch and Meade (2016) found that while the N170 latencies to words and pseudowords did not differ in typically developing 3rd and 5th graders, pseudowords elicited longer latency responses than words in 4th graders. Lastly, studies in young adults showed no difference between the N170 response latencies to words and pseudo-homophones (Taha and Khateb, 2013), words, and pseudowords (Coch and Meade, 2016) or pseudowords and non-words (Coch and Meade, 2016).

##### Lateralization

Eighteen studies investigated the lateralization of the N170 response in word-like conditions. In a study comparing different reading ability groups of school-aged children, Hasko et al. (2013) found no differences between TD and DRD groups, as both groups showed a bilateral distribution of the N170 to words and non-words. Similar bilateral response distribution was reported for pseudowords in TD children (Jucla et al., 2010; Hasko et al., 2013) and in DRD children (Jucla et al., 2010; Kast et al., 2010). In studies on young adults, Araújo et al. (2015) found no differences between TD and DRD groups, wherein pseudo-homophones generated a larger N170 than words and pseudowords over the right hemisphere but showed no differences in the N170 between the word and word-like conditions over the left hemisphere. Meanwhile, the N170 response to pseudowords showed a left-lateralized distribution trend in TD adults (Mahé et al., 2012, 2013) but showed the reverse for DRD adults (Mahé et al., 2012).

The remaining studies only reported lateralization on TD readers in one age group: one study in pre-literate children, two in school-aged children, and nine in young adults. For pre-literate children, the N170 response to pseudowords showed bilateral distribution, similar to the word condition in pre-literate (Zhao et al., 2018) and school-aged children (Eberhard-Moscicka et al., 2015). However, non-words showed a left-lateralized distribution of the N170 (Pleisch et al., 2019). In young adults, most of the studies showed left-lateralization of the N170 for pseudowords or pseudo-characters (Simon et al., 2007; Cao and Zhang, 2011; Lin et al., 2011; Wei et al., 2018), pseudo-homophones (Taha and Khateb, 2013) and non-words (Okumura et al., 2015; Uno et al., 2017), whereas one showed bilateral activation of the N170 for non-words (Okumura et al., 2014). In addition, Faísca et al. (2019) compared words versus pseudowords and found that while the N170 responses to words were more negative over the left recording sites than to pseudowords, no difference in the N170 was present over the right (Faísca et al., 2019). Meanwhile, other studies showed no difference in the N170 between words and pseudowords over the left hemisphere (Simon et al., 2007; Wei et al., 2018).

## Discussion

This work is the first systematic review that assimilated existing research on the N170 response to words (N170w) in individuals with and without reading difficulties. Out of seven databases, 69 peer-reviewed studies were included, of which the majority was conducted in adults, followed by school-aged children and pre-readers, mainly in German-, English- or Chinese-speaking populations. Our main goal was to synthesize findings on the differences in the N170w amplitude, latency, and lateralization and to capture the typical and atypical development of the N170w by comparing different age groups, namely, pre-literate children (3–6 years old), school-aged/literate children (7–11 years old) and young adults (18–35 years old). Aside from this, we aimed to shed light on the assumed fine-tuning of the emerging print expertise shown by the N170 by comparing the N170w with those recorded in word-like conditions across different studies. Lastly, we compared the ERP methods used across studies. Here, our main objective was to provide an overview of various paradigms and recording systems used in N170 research in reading.

### Comparison of N170w in Typical and Atypical Readers

Results on the N170w amplitude illustrate that most TD readers, particularly adults, show a larger, more negative N170w than impaired readers. This larger and more negative N170w in typical readers can be explained by a more efficient visual orthographic processing, e.g., expertise in print. It has been interpreted in the reviewed literature to indicate effective orthographic processing during the prelexical stage (Simon et al., 2007; Dujardin et al., 2011; González-Garrido et al., 2014) as well as an efficient specialization enhanced by exposure to print and successful reading acquisition *via* efficient learning and conversion of letter-sound correspondences (Brem et al., 2013; Zhao et al., 2015). Here, poorer reading performance of the DRD/PR groups compared to TD has been interpreted as a consequence of insufficient visual tuning or identification of print or word forms, which continued as persistent weakness in adulthood. Based on the reviewed literature, this can imply a slower, inconsistent orthographic processing (Savill and Thierry, 2011a; Waldie et al., 2012), lower general reactivity to orthographic stimuli (Maurer et al., 2005; Savill and Thierry, 2011a), impairment of visual plasticity exclusive to print at the beginning of reading acquisition (Maurer et al., 2007), deficient processing in visual recognition cortical areas (Kast et al., 2010) or unconventional specialization of the responsible brain networks (Mahé et al., 2012, 2013). Different cognitive domains have also been suggested as responsible modulators of the orthographic specialization reflected by the N170w. One is the inefficient attention allocation system, as shown by the P1 ERP component, suggesting the importance of domain-general functions related to visual processing (Korinth et al., 2012; Korinth and Breznitz, 2014).

The N170w latency findings suggest similar processing time, e.g., similar latencies in TD and DRD groups, or longer latency in controls compared to DRD in childhood. However, in adulthood, findings consistently report a longer latency for DRD than controls. This result is interpreted in the literature as less efficient processing of orthographic cues in dyslexic participants (Savill and Thierry, 2011a). Moreover, this delayed processing of words may be associated with neurobiological deviations reflected in the electrophysiological correlates, here the N170w, in impaired readers. Differences in the structural connectivity, atypical hemispheric asymmetry, or processing differences shown by EEG band power and coherence could possibly show these assumed neurobiological differences (e.g., Arns et al., 2007; Dhar et al., 2010; Fraga González et al., 2016a, 2018). In Waldie et al. (2012) study, event-related brain potentials and EEG coherence, measuring the neural synchrony, were investigated in late-proficient bilingual, dyslexic, and control adult participants performing a lexical task. While higher synchrony was found between hemispheres in the gamma range in the dyslexic group, the same was found in the theta range compared to the control group. In addition, the higher between-hemisphere synchrony was accompanied by lower amplitude N170w in the dyslexic group. The authors interpreted their findings as an asynchrony of neuronal activity at the crucial moment when word forms need to be distinguished. However, the EEG/MEG connectivity studies available on TD-DRD comparisons yield inconclusive results and should further be examined in future studies.

Specialization of print, part of the reading and language network (McCandliss et al., 2003; Dehaene et al., 2010), is typically reported to be left-lateralized in typical readers and bilateral in impaired readers. This left lateralization is thought to be driven by phonological processing, referred to as the phonological mapping hypothesis suggested by Maurer and McCandliss (2008). Evidence for this phonological mapping hypothesis has been specially found for languages that use grapheme-phoneme conversion rules (i.e., alphabetic languages) but has been challenged in studies that used logographic or syllabic languages using lexical morphemes (Maurer and McCandliss, 2008; more discussion, see Linguistic Factors Section). The core idea of this hypothesis was that print processing in the visual cortex underwent left lateralization due to the left-lateralized phonological processing (Price et al., 1997; Rumsey et al., 1997). Although beyond the scope of this review, this early theory might correspond to findings on a left-lateralized hemodynamic activity during visual word recognition tasks (Brem et al., 2009; Maurer et al., 2011; Pleisch et al., 2019). Several neuroimaging studies identified the left ventral occipitotemporal cortex, referred to as the Visual Word Form area (VWFA), as a critical structure for fluent and efficient word recognition (Cohen et al., 2000; McCandliss et al., 2003; Dehaene et al., 2010; Coch and Meade, 2016). This argument is beyond the scope of this review, however for more discussion, we refer to Cohen et al. (2000) and Chen et al. (2019).

The preferential activation to print in the left ventral occipitotemporal cortex has been attributed to successful grapheme-phoneme learning and mapping when formal reading instruction begins (Brem et al., 2010; Karipidis et al., 2017; Pleisch et al., 2019), as well as visual or script familiarity (Brem et al., 2013) or higher word knowledge in pre-literate children, thus highlighting the key role of reading exposure (Li et al., 2013). However, for school-aged children, some studies have reported a more right-lateralized N170w (or reduced left-lateralization of N170w) for typical readers (Fraga González et al., 2014, 2016b; Zhao et al., 2014), which may contradict the general assumption that successful grapheme-phoneme correspondences indicate left-lateralization. The authors indicated that this reduced left-lateralization of N170w for typical readers (Fraga González et al., 2014, 2016b; Zhao et al., 2014) could be due to (a) specialization of the visual word form area, implying a successful lexical access and whole-word level specialization (Fraga González et al., 2014), (b) more automatized reading in typical readers (Maurer et al., 2006; Fraga González et al., 2014) or (c) employment of attentional strategies in orthographic word decoding than processing phonology or semantic information (Fraga González et al., 2014, 2016b). This slight right-lateralization was also reported for pre-literate children that were later classified as poor readers, which can be attributed but not limited to visual familiarity to letters (Brem et al., 2013). This result is due to the non-reading preschoolers but with high letter knowledge as their sample, indicating that exposure may have helped it develop even before reading instruction starts (Maurer et al., 2005; Brem et al., 2013). Aside from this, Brem et al. (2010) found that in pre-literate children with eventual poor reading outcomes, this right-lateralized negativity can be attributed to possible differences in print processing strategies which can be modulated by attention (i.e., focusing on whole-word associations strategy than using letter-sound correspondences). Lastly, some studies on TD school-aged children also reported bilateral activation, which was interpreted as a delayed or missing automatization or an incomplete development of print sensitivity (Hasko et al., 2013). Likewise, this bilateral activation was found in impaired readers, in pre-literate age, childhood, and adulthood, which might indicate a failure to exhibit automatic grapheme-phoneme conversion needed for skilled reading, and is typically mastered through increased exposure to print and continuous reading experience (Brem et al., 2010; Karipidis et al., 2017; Pleisch et al., 2019).

### Development of the N170w

Generally, results indicate an amplitude decrease of the N170w with age; thus, less negative N170w amplitudes have been reported in adults than children. This amplitude decrease has been suggested to be related to more reading experience (Brem et al., 2010; Karipidis et al., 2017; Pleisch et al., 2019) and fluency gains (Fraga González et al., 2016b). Studies that looked into young and old subgroups within school-aged children have consistently found that larger N170w was elicited in younger groups than in their older counterparts, reflecting a higher print tuning in the early phase of reading acquisition (Maurer et al., 2007). This developmental course across studies adheres to the suggested inverted U-shaped development of print tuning (Maurer et al., 2006; Fraga González et al., 2014; Pleisch et al., 2019) found as evident in the three age groups included in our review. Pre-literates showed low N170w amplitude due to non-exposure (Maurer et al., 2006), which increased upon the start of reading instruction mainly during the first two years of learning to read and then leveled off around the second to fifth grade (Maurer et al., 2011) continuing to decrease to adulthood as a result of increased exposure with a consequence of enhanced print expertise (Fraga González et al., 2014). Maurer et al. (2006) argued that such plastic reorganization of the brain for print could not be due to general maturation, as this would lead to an increased N170 for both words and matched symbols. N170w latencies showed a characteristic developmental trajectory, with adults having an earlier onset than school-aged and pre-literate children. This result was interpreted to reflect automatization after becoming an expert reader (Maurer et al., 2006). Lastly, the N170w of typical readers changed its bilateral distribution to a dominant left-hemispheric presence throughout development, whereas this response was mainly right-lateralized for younger poor readers and continuously remained in a bilateral distribution. This N170w left-lateralization throughout development can be attributed to the synchrony of orthographic and phonological correspondences as reading expertise improves, indicating a word reading automaticity (Maurer and McCandliss, 2008).

### Word vs. Word-Like Conditions

Results looking into the differences in amplitude, latency, and lateralization of the N170 elicited in word, and word-like conditions (pseudowords, pseudo-homophones, non-words) report huge variability across studies comparing TD and DRD/PR groups. These comparisons investigated how lexicality effects might be involved in the processes giving rise to the N170 component. Some studies referred to the changes found as the result of fine-tuning, which involved different processing for words compared to word-like stimuli. These changes could possibly be influenced by early lexical activation (Mahé et al., 2013), usually occurring at the late interval of the N1 ERP component (Eberhard-Moscicka et al., 2016). Unlike the early maturation for print upon reading instruction in children (Maurer et al., 2007; Brem et al., 2013; Eberhard-Moscicka et al., 2015), the emergence of selective responses to word forms most likely follows a prolonged development since it would require higher reading abilities to delineate different types of word forms (Centanni et al., 2018; Pleisch et al., 2019). In this case, this could partly explain different results across studies described below when comparing word and word-like conditions.

Differences in the fine-tuning are thought to relate to processes reflected by the later N1 associated with orthographic regularity or pronunciability, thus expecting pseudowords to elicit a larger N170 than non-words for typically developing children (Zhao et al., 2014). Failure for DRD/PR individuals to catch these pseudoword-non-word differences can be attributed to impaired sublexical orthographic processing, which may entail less sensitivity to letter positioning and sequences (Araújo et al., 2015). Furthermore, differences between words and pseudowords are brought by non-automatized grapheme-phoneme mapping, thus reflecting non-generalization of N170w specialization to pseudowords (Maurer and McCandliss, 2008). Some studies showed a right-lateralized lexicality effect in the DRD/PR group, indicating a right hemisphere overactivation typical for the DRD population, which is negatively correlated with reading skills (Shaywitz et al., 1998, 2002; Mahé et al., 2012). However, aside from these possible scenarios, other factors that could explain the variations found in results across studies can be attributed to a lack of lexical access (Korinth et al., 2012), variations in stimulus material, i.e., linguistic and non-linguistic stimuli (Barber and Kutas, 2006; Hasko et al., 2013), task design and demands, i.e., implicit and explicit tasks (Bentin et al., 1999; Faísca et al., 2019), limited reading experience (Kast et al., 2010; Hasko et al., 2013) or linguistic variables (Bentin et al., 1999; Pegado et al., 2014; further discussed in Section Linguistic factors).

With regards to lateralization in TD readers, words and pseudowords showed a similar bilateral distribution of the N170 in pre-literate age and childhood, which could be affected by the degree as to which reading stage they were in, wherein this case, these children might not have enough exposure yet to have developed print expertise or automatized grapheme-phoneme mapping (Brem et al., 2010; Karipidis et al., 2017; Pleisch et al., 2019). An alternative explanation would be the task demands or linguistic variables (Eberhard-Moscicka et al., 2015). Most studies using word-like conditions in TD adults showed left-lateralized responses to pseudowords, arguing that print specialization generalizes from words to well-ordered letter strings (Maurer and McCandliss, 2008; Dujardin et al., 2011). For alphabetic scripts, this is probably the result of the recruitment of phonology in the successful activation of grapheme-phoneme mapping, known as the phonological mapping hypothesis (Maurer and McCandliss, 2008), whereas, for logographic scripts, it must be primarily based on orthographic processing rather than phonology due to arbitrary sound-graphic correspondences or the reliance on morpheme structures (Zhou et al., 2009; Lin et al., 2011). However, the interpretation for left-lateralization of pseudowords should be taken into caution depending on the orthographic depth of the language involved as those with inconsistent grapheme to phoneme mappings (i.e., opaque orthography) complicates the automaticity and might therefore lead to non-left-lateralization (Maurer and McCandliss, 2008).

### Linguistic Factors

It has been long contested whether alphabetic vs. logographic languages are processed differently in the brain, and a few studies tried to investigate this in relation to the N170 (Wong et al., 2005; Maurer et al., 2008b; Cao et al., 2011; Lin et al., 2011; Qin et al., 2016). The authors aimed to answer whether the type of orthographic script modulates the lateralization of print specialization or whether it is entirely dependent on script familiarity of the participants involved in the experiments. From our systematic search, studies focusing on alphabetic scripts, mainly Latin scripts, mostly found evidence for a left-lateralized N170w (e.g., Maurer et al., 2005; Dujardin et al., 2011; Mahé et al., 2012, 2013; Dundas et al., 2014; González-Garrido et al., 2014; Araújo et al., 2015; Collins et al., 2017). Studies conducted in Chinese or Japanese found that logographic or syllabic scripts, revealed a left-lateralized N170 response to their characters as well (Maurer et al., 2008b; Cao et al., 2011; Yum et al., 2011; Qin et al., 2016; Wei et al., 2018; Xue et al., 2019). These findings collectively suggest that left-lateralization develops through reading expertise and visual form familiarity even in languages without direct grapheme-phoneme mapping.

Aside from the type of the scripts, the orthographic depth of a language (i.e., the consistency in which spelling is mapped onto sounds; Schmalz et al., 2015) has also been investigated as a potential modulator for the N170w, with transparent languages having more direct correspondences and opaque languages having less direct correspondences. According to the classification of European languages of Seymour et al. (2003) and the included papers using Asian languages, the reviewed studies showed some orthographic diversity with 52% of the sample investigating deep orthography languages (e.g., English, French, Chinese), 33% including shallow orthographies (e.g., German, Japanese) and the remaining 15% including languages that were medium transparent (e.g., Dutch, Portuguese, Arabic). Most studies in deep orthography showed a more negative N170w for controls than their reading-impaired counterparts (in English: Savill and Thierry, 2011a,b; Waldie et al., 2012; Collins et al., 2017; in French: Jucla et al., 2010; Mahé et al., 2012, 2013; in Hebrew: Korinth and Breznitz, 2014), whereas those categorized in the middle showed the opposite; a larger N170w in impaired readers compared to controls (Fraga González et al., 2014, 2016b; van Setten et al., 2019). Split results have also been noted in studies with shallow orthography depending on the age group. A more detailed investigation of this is beyond the scope of this review. However, it could pose another question for future studies on how N170w is affected by lexical vs. non-lexical reading routes, hence an area of further exploration regarding the differences between coarse and fine-tuning of N170 across different orthographies.

### Methodological Considerations

The studies discussed in the current review have used a variety of experimental designs, yielding many different variables across experiments. Before the measured raw signal can be analyzed, it has to undergo a series of preprocessing operations, such as re-referencing, offline filtering, correcting or rejecting artifacts, which might in themselves influence ERP outcomes. Moreover, included studies have shown a considerable disparity on how and where ERPs are quantified. Most studies have used GFP or previous literature to determine the N170w time window, using the mean activity within this time window or detecting the most extreme amplitude value. Despite Picton et al. (2000) giving guidelines for reporting the results of ERP studies, a significant amount of our reviewed studies did not achieve a holistic reporting of all essential aspects necessary to compare the methodology of ERP research. Studies mostly focused on P7, P8, and O1 electrode sites but sometimes were even spread further for temporal sites such as T7 and T8. Moreover, a significant variety was found across studies in using single electrode activity or mean activity within an electrode cluster. Despite the choice of cluster or single electrode analyses, the recording location of the electrodes should be considered, as it diversifies the findings within/across studies as the scalp position of the electrode might differ from the template position. Digitization of electrode position or clear deviation description is advisable (Picton et al., 2000). Furthermore, the diversity of the EEG acquisition systems calls for an evaluation of the effect of the acquisition system as a contributing variable for N170w amplitude. More care should be given to the amplifier specifications and online filtering of the recordings. Even in offline filtering, the filter choice should be described in detail (i.e., backward, forward, zero phases), and the same is valid for the high pass and low pass values. Most articles delivered the voltage level applied for thresholds used in artifact rejections and stated other artifact rejection methods, though, were inconsistent in reporting the final number of trials used for averaging. The final number of trials per condition is an essential factor to report, even to evaluate the amplitude measure (peak, mean over time window) used to extract the N170w value (Picton et al., 2000). Most of the reviewed studies gave an illustration of ERP waveforms, although the labeling of graphs did not follow a convention. Therefore, it is advisable to pay attention to explicit labeling. Crucial to be aligned across studies is the reporting of statistical tests, their outcomes, and effect sizes, especially descriptive values of the N170w amplitude and latency were lacking in many studies. It should be considered as good practice to provide a satisfactory amount of statistical information in order to help the reader to understand the full scope of calculations, as well as enabling to compute, e.g., effect sizes if wanted. For a more comprehensive guide on statistical reporting in brain research, see Gross et al. (2013). For more comprehensive discussion and guidance on ANOVA application in ERP research, we refer to Dien (2017).

Substantial heterogeneity was found across experimental paradigms. Most studies used a repetition-detection task or a lexical decision task, with a combination of words, pseudowords, and symbols to examine the N170w. Different outcomes of the N170w, particularly its left-lateralization, among age and reading-ability groups might vary due to external factors such as stimuli and tasks/experimental designs (Maurer et al., 2007; van Setten et al., 2019). Faísca et al. (2019) mentioned that while early lexical effects (more negative N170w than pseudowords) were evident in implicit tasks (e.g., one-back repetition task) on adults, this result could not be replicated in explicit tasks (e.g., delayed reading aloud). In contrast, Maurer et al. (2005) noted that explicit linguistic tasks showed more sensitivity to lexical differences than implicit tasks; however, the study also highlighted exceptions, even though scarce, in which linguistic characteristics of the stimuli can affect N170w. Regardless of the two different points of view, the N170w can be seen as dependent on task demands representing a difference in susceptibility to top-down processes based on the tasks' goal, especially when investigating the fine-tuning component, which is related to early lexicality effects (Faísca et al., 2019). It is perceived that tasks that require low-level visual recognition (e.g., repetition detection tasks) may have elicited a much more automatic reading for words than pseudowords, whereas, for conscious linguistic tasks, a focus on the grapheme to phoneme decoding may have taken place (Maurer et al., 2005; Eberhard-Moscicka et al., 2016; Faísca et al., 2019). Moreover, left-lateralization of N170w is linked as well with the type of processing strategies or attentional allocation during the early phase of reading acquisition (Maurer et al., 2010; Faísca et al., 2019), but as reading expertise is enhanced with age, the left-lateralization becomes automatic and less susceptible to the attention and task demands (Strijkers et al., 2011; Faísca et al., 2019).

This flexibility in experimental designs and data analysis is a common target of criticism, as it inflates the chance of false positives and complicates the comparison of findings across studies. Several authors attempted to provide publication guidelines to facilitate methodological transparency (Picton et al., 2000; Keil et al., 2014; Clayson et al., 2019; Paul et al., 2021). It remains an educational process for researchers, as such information is crucial to assess the quality of research and ensure that enough information is available to undertake replication studies. One way to overcome these issues is study preregistration, a locked plan containing a study hypothesis, methodology, and data analysis plan (Paul et al., 2021). Enhancing the use of pre-registration in interaction with the common alignment of EEG data analyses as an approach to overcome the reproducibility and comparability of EEG analyses could be considered.

### Limitations

Studies on pre-literate children in this review are generally scarcer than the other age groups. The reported results for pre-literate children are taken from three studies only, thus giving limited power in driving solid conclusions due to the limited sample size. Aside from this, different EEG acquisition systems, pre-processing steps, and experimental designs have been utilized in the studies included in the review, offering substantial heterogeneity. Variations in the EEG preprocessing steps, such as different filtering values, can also affect direct comparisons.

Reporting of results seemed incomplete as most of the papers did not report mean amplitude or latency values; hence, no claims can be made about the contribution of the proposed moderators. Our review synthesizes the results of the included studies qualitatively with the inclusion of descriptive statistics for some variables, as it was not possible to obtain enough effect sizes for the computation of funnel plots from the given data of the papers included in this systematic review. Most studies did not provide complete statistical information to calculate these effect sizes in their manuscripts. Thus, an argument can be made that this systematic review represents literature with a publication bias, as we only included peer-reviewed studies and did not access gray literature. To address these limitations, a meta-analysis is highly called upon to provide a more comprehensive picture of the N170w.

Due to our strictly focused search on N1/N170 in words and reading disorders in children or adults, it is unavoidable that we may have missed relevant studies on N170 (e.g., Qin et al., 2016) that did not use all of the combinations stated in our search strategy (e.g., no mention of keywords “word”, AND “develop^*^”). In this case, these articles did not appear in our search and thus, were not included in this review. Alongside, this review only included studies published before mid-January of 2021; thus, all new publications after this period, even though they would fit the criteria, are not included and analyzed here. Another limitation in this domain is excluding combined data approaches, such as fixation-related potentials. A strict standpoint is taken on combined data studies, as one aspect of the presented systematic review was the methodological consideration of ERP research in word recognition, and combined data analyses commonly go beyond the methodological scope of conventional EEG research, thus are not comparable, especially about the perception of words. For a recent review of fixation-related potentials and reading, one can explore Degno and Liversedge (2020).

Lastly, dyslexia screening and assessment tools varied widely across the included studies, yielding different criteria to classify participants as reading impaired or typical reader. This variation might be important to consider in comparing results due to the possibility of different degrees of reading difficulties, as well the potential inclusion of different DRD subtypes. Previous studies successfully identified subtypes of DRD using learning algorithms such as mixed modeling (Torppa et al., 2007), latent profile analysis (Wolff, 2010) and confirmatory latent profile analysis (Niileksela and Templin, 2019). Although it would be interesting to see how DRD subtypes affect N170w development, this might be challenging in brain research due to lower sample sizes. Only two studies in the current review looked into subtypes; One study looked into specific difficulties in reading and spelling (Kemény et al., 2018) but did not find significant differences between the reading and spelling deficit groups, and Dujardin et al. (2011) identified dyslexia subgroups on the basis of N170 but not on the basis of their reading related skills as those did not yield a significant difference.

## Conclusion

This review provides a more comprehensive overview of the development of the N170w across age groups (pre-literate age, school-aged and adulthood) and reading abilities (typically developing, developmental reading disorders/ developmental dyslexia/poor readers), as well as the response of N170 between word and word-like stimuli. Lastly, we discussed theoretical and methodological differences and challenges in the field to guide future research. Results showed that in adult studies, N170w amplitude is more negative in the controls than the poor readers, although mixed results were reported for children with varying reading ability. N170w lateralization is also in question, as left-lateralization is more straightforwardly reported in typical adults but still variable during childhood. Lastly, N170w vs. other word-like conditions gave mixed results across studies, depending on the investigated hemisphere, stimuli and tasks employed, as well as linguistic variables.

## Data Availability Statement

The original contributions presented in the study are included in the article/Supplementary Material, further inquiries can be directed to the corresponding author/s.

## Author Contributions

KA and VC: conceptualization. KA, AT, CV, JT, PL, and VC: protocol writing, revisions, and writing—revision and editing. KA, AT, and CV: database search, synthesis of results, analysis, and writing—original draft. All authors contributed to the article and approved the submitted version.

## Funding

This research was funded by the Neo-PRISM-C project (European Union Horizon 2020 Program, H2020-MSCA-ITN-2018) under the Marie Skłodowska-Curie Innovative Training Network (Grant Agreement No. 813546).

## Conflict of Interest

The authors declare that the research was conducted in the absence of any commercial or financial relationships that could be construed as a potential conflict of interest.

## Publisher's Note

All claims expressed in this article are solely those of the authors and do not necessarily represent those of their affiliated organizations, or those of the publisher, the editors and the reviewers. Any product that may be evaluated in this article, or claim that may be made by its manufacturer, is not guaranteed or endorsed by the publisher.

## References

[B1] Araújo S. Bramão I. Faísca L. Petersson K. M. Reis A. (2012). Electrophysiological correlates of impaired reading in dyslexic pre-adolescent children. Brain Cogn. 79, 79–88. 10.1016/j.bandc.2012.02.010 22466501

[B2] Araújo S. Faísca L. Bramão I. Reis A. Petersson K. M. (2015). Lexical and sublexical orthographic processing: an ERP study with skilled and dyslexic adult readers. Brain Lang. 141, 16–27. 10.1016/j.bandl.2014.11.007 25528285

[B3] Arns M. Peters S. Breteler R. Verhoeven L. (2007). Different brain activation patterns in dyslexic children: evidence from EEG power and coherence patterns for the double-deficit theory of dyslexia. J. Integr. Neurosci. 6, 175–190. 10.1142/S0219635207001404 17472228

[B4] Bakos S. Landerl K. Bartling J. Schulte-Körne G. Moll K. (2018). Neurophysiological correlates of word processing deficits in isolated reading and isolated spelling disorders. Clin. Neurophysiol. 129, 526–540. 10.1016/j.clinph.2017.12.010 29353181

[B5] Barber H. A. Kutas M. (2006). Interplay between computational models and cognitive electrophysiology in visual word recognition. Brain Res. Rev. 53, 98–123. 10.1016/j.brainresrev.2006.07.002 16905196

[B6] Bentin S. Allison T. Puce A. Perez E. McCarthy G. (1996). Electrophysiological studies of face perception in humans. J. Cogn. Neurosci. 8, 551–565. 10.1162/jocn.1996.8.6.551 20740065 PMC2927138

[B7] Bentin S. Mouchetant-Rostaing Y. Giard M. H. Echallier J. F. Pernier J. (1999). ERP manifestations of processing printed words at different psycholinguistic levels: time course and scalp distribution. J. Cogn. Neurosci. 11, 235–260. 10.1162/089892999563373 10402254

[B8] Brem S. Bach S. Kucian K. Kujala J. V. Guttorm T. K. Martin E. . (2010). Brain sensitivity to print emerges when children learn letter–speech sound correspondences. Proc. Natl. Acad. Sci. 107, 7939–7944. 10.1073/pnas.0904402107 20395549 PMC2867899

[B9] Brem S. Bach S. Kujala J. V. Maurer U. Lyytinen H. Richardson U. . (2013). An electrophysiological study of print processing in kindergarten: the contribution of the visual N1 as a predictor of reading outcome. Dev. Neuropsychol. 38, 567–594. 10.1080/87565641.2013.828729 24219696

[B10] Brem S. Bucher K. Halder P. Summers P. Dietrich T. Martin E. . (2006). Evidence for developmental changes in the visual word processing network beyond adolescence. NeuroImage 29, 822–837. 10.1016/j.neuroimage.2005.09.023 16257546

[B11] Brem S. Halder P. Bucher K. Summers P. Martin E. Brandeis D. (2009). Tuning of the visual word processing system: distinct developmental ERP and fMRI effects. Hum. Brain Mapp. 30, 1833–1844. 10.1002/hbm.20751 19288464 PMC6871060

[B12] Cao F. Rickles B. Vu M. Zhu Z. Chan D. H. L. Harris L. N. . (2013). Early stage visual-orthographic processes predict long-term retention of word form and meaning: a visual encoding training study. J. Neurolinguistics 26, 440–461. 10.1016/j.jneuroling.2013.01.003 23798804 PMC3689543

[B13] Cao X. Li S. Zhao J. Lin S. Weng X. (2011). Left-lateralized early neurophysiological response for Chinese characters in young primary school children. Neurosci. Lett. 492, 165–169. 10.1016/j.neulet.2011.02.002 21310213

[B14] Cao X.-H. Zhang H.-T. (2011). Change in subtle N170 specialization in response to Chinese characters and pseudocharacters. Percept. Mot. Skills 113, 365–376. 10.2466/04.22.24.28.PMS.113.5.365-376 22185051

[B15] Centanni T. M. Norton E. S. Park A. Beach S. D. Halverson K. Ozernov-Palchik O. . (2018). Early development of letter specialization in left fusiform is associated with better word reading and smaller fusiform face area. Dev. Sci. 21, e12658. 10.1111/desc.12658 29504651 PMC6115291

[B16] Chen L. Wassermann D. Abrams D. A. Kochalka J. Gallardo-Diez G. Menon V. (2019). The visual word form area (VWFA) is part of both language and attention circuitry. Nat Commun 10, 5601. 10.1038/s41467-019-13634-z 31811149 PMC6898452

[B17] Clayson P. E. Carbine K. A. Baldwin S. A. Larson M. J. (2019). Methodological reporting behavior, sample sizes, and statistical power in studies of event-related potentials: barriers to reproducibility and replicability. Psychophysiology 56. 10.1111/psyp.13437 31322285

[B18] Coch D. Meade G. (2016). N1 and P2 to words and wordlike stimuli in late elementary school children and adults. Psychophysiology 53, 115–128. 10.1111/psyp.12567 26473497 PMC4715684

[B19] Cohen L. Dehaene S. Naccache L. Lehéricy S. Dehaene-Lambertz G. Hénaff M.-A. . (2000). The visual word form area: spatial and temporal characterization of an initial stage of reading in normal subjects and posterior split-brain patients. Brain 123, 291–307. 10.1093/brain/123.2.291 10648437

[B20] Collins E. Dundas E. Gabay Y. Plaut D. C. Behrmann M. (2017). Hemispheric organization in disorders of development. Vis. Cogn. 25, 416–429. 10.1080/13506285.2017.1370430 30464702 PMC6241318

[B21] Curzietti M. Bonnefond A. Staub B. Vidailhet P. Doignon-Camus N. (2017). The effects of age on visual expertise for print. Brain Lang. 169, 48–56. 10.1016/j.bandl.2017.03.001 28327370

[B22] Davis C. P. Libben G. Segalowitz S. J. (2019). Compounding matters: event-related potential evidence for early semantic access to compound words. Cognition 184, 44–52. 10.1016/j.cognition.2018.12.006 30557749

[B23] Degno F. Liversedge S. P. (2020). Eye movements and fixation-related potentials in reading: a review. Vision 4, 11. 10.3390/vision4010011 32028566 PMC7157570

[B24] Dehaene S. Pegado F. Braga L. W. Ventura P. Filho G. N. Jobert A. . (2010). How learning to read changes the cortical networks for vision and language. Science 330, 1359–1364. 10.1126/science.1194140 21071632

[B25] Dhar M. Been P. H. Minderaa R. B. Althaus M. (2010). Reduced interhemispheric coherence in dyslexic adults. Cortex 46, 794–798. 10.1016/j.cortex.2009.09.006 19822316

[B26] Dien J. (2017). Best practices for repeated measures ANOVAs of ERP data: reference, regional channels, and robust ANOVAs. Int. J. Psychophysiol. 111, 42–56. 10.1016/j.ijpsycho.2016.09.006 27622381

[B27] Dujardin T. Etienne Y. Contentin C. Bernard C. Largy P. Mellier D. . (2011). Behavioral performances in participants with phonological dyslexia and different patterns on the N170 component. Brain Cogn. 75, 91–100. 10.1016/j.bandc.2010.10.006 21094575

[B28] Dundas E. M. Plaut D. C. Behrmann M. (2014). An ERP investigation of the co-development of hemispheric lateralization of face and word recognition. Neuropsychologia 61, 315–323. 10.1016/j.neuropsychologia.2014.05.006 24933662 PMC4251456

[B29] Eberhard-Moscicka A. K. Jost L. B. Fehlbaum L. V. Pfenninger S. E. Maurer U. (2016). Temporal dynamics of early visual word processing – early versus late N1 sensitivity in children and adults. Neuropsychologia 91, 509–518. 10.1016/j.neuropsychologia.2016.09.014 27659875

[B30] Eberhard-Moscicka A. K. Jost L. B. Raith M. Maurer U. (2015). Neurocognitive mechanisms of learning to read: print tuning in beginning readers related to word-reading fluency and semantics but not phonology. Dev. Sci. 18, 106–118. 10.1111/desc.12189 24863157

[B31] Emmorey K. Midgley K. J. Kohen C. B. Sehyr Z. S. Holcomb P. J. (2017). The N170 ERP component differs in laterality, distribution, and association with continuous reading measures for deaf and hearing readers. Neuropsychologia 106, 298–309. 10.1016/j.neuropsychologia.2017.10.001 28986268 PMC5694363

[B32] Faísca L. Reis A. Araújo S. (2019). Early brain sensitivity to word frequency and lexicality during reading aloud and implicit reading. Front. Psychol. 10, 830. 10.3389/fpsyg.2019.00830 31031684 PMC6470259

[B33] Feuerriegel D. Churches O. Hofmann J. Keage H. A. D. (2015). The N170 and face perception in psychiatric and neurological disorders: A systematic review. Clin. Neurophysiol. 126, 1141–1158. 10.1016/j.clinph.2014.09.015 25306210

[B34] Fraga González G. Pleisch G. Di Pietro S. V. Neuenschwander J. Walitza S. Brandeis D. . (2021). The rise and fall of rapid occipito-temporal sensitivity to letters: transient specialization through elementary school. Dev. Cogn. Neurosci. 49, 100958. 10.1016/j.dcn.2021.100958 34010761 PMC8141525

[B35] Fraga González G. Smit D. J. A. van der Molen M. J. W. Tijms J. Stam C. J. de Geus E. J. C. . (2018). EEG resting state functional connectivity in adult dyslexics using phase lag index and graph analysis. Front. Hum. Neurosci. 12, 341. 10.3389/fnhum.2018.00341 30214403 PMC6125304

[B36] Fraga González G. Van der Molen M. J. W. Žarić G. Bonte M. Tijms J. Blomert L. . (2016a). Graph analysis of EEG resting state functional networks in dyslexic readers. Clin. Neurophysiol. 127, 3165–3175. 10.1016/j.clinph.2016.06.023 27476025

[B37] Fraga González G. Žarić G. Tijms J. Bonte M. Blomert L. Leppänen P. . (2016b). Responsivity to dyslexia training indexed by the N170 amplitude of the brain potential elicited by word reading. Brain Cogn. 106, 42–54. 10.1016/j.bandc.2016.05.001 27200495

[B38] Fraga González G. Žarić G. Tijms J. Bonte M. Blomert L. van der Molen M. W. (2014). Brain-potential analysis of visual word recognition in dyslexics and typically reading children. Front. Hum. Neurosci. 8, 474. 10.3389/fnhum.2014.00474 25071507 PMC4075352

[B39] González-Garrido A. A. Gómez-Velázquez F. R. Rodríguez-Santillán E. (2014). Orthographic recognition in late adolescents: an assessment through event-related brain potentials. Clin. EEG Neurosci. 45, 113–121. 10.1177/1550059413489975 24043221

[B40] Gross J. Baillet S. Barnes G. R. Henson R. N. Hillebrand A. Jensen O. . (2013). Good practice for conducting and reporting MEG research. Neuroimage 65, 349–363. 10.1016/j.neuroimage.2012.10.001 23046981 PMC3925794

[B41] Hasko S. Groth K. Bruder J. Bartling J. Schulte-Körne G. (2013). The time course of reading processes in children with and without dyslexia: an ERP study. Front. Hum. Neurosci. 7, 570. 10.3389/fnhum.2013.00570 24109444 PMC3791381

[B42] Herrmann C. S. Knight R. T. (2001). Mechanisms of human attention: event-related potentials and oscillations. Neurosc. Biobehav. Rev. 25, 465–476. 10.1016/S0149-7634(01)00027-6 11595268

[B43] Jucla M. Nenert R. Chaix Y. Demonet J.-F. (2010). Remediation effects on N170 and P300 in children with developmental dyslexia. Behav. Neurol. 22, 121–129. 10.1155/2010/913692 20595744 PMC5434325

[B44] Karipidis I. Pleisch G. Röthlisberger M. Hofstetter C. Dornbierer D. Stämpfli P. . (2017). Neural initialization of audiovisual integration in prereaders at varying risk for developmental dyslexia. Hum. Brain Mapp. 38, 1038–1055. 10.1002/hbm.23437 27739608 PMC6866885

[B45] Kast M. Elmer S. Jancke L. Meyer M. (2010). ERP differences of pre-lexical processing between dyslexic and non-dyslexic children. Int. J. Psychophysiol. 77, 59–69. 10.1016/j.ijpsycho.2010.04.003 20420862

[B46] Keil A. Debener S. Gratton G. Junghöfer M. Kappenman E. S. Luck S. J. . (2014). Committee report: publication guidelines and recommendations for studies using electroencephalography and magnetoencephalography: guidelines for EEG and MEG. Psychophysiology 51, 1–21. 10.1111/psyp.12147 24147581

[B47] Kemény F. Banfi C. Gangl M. Perchtold C. M. Papousek I. Moll K. . (2018). Print-, sublexical and lexical processing in children with reading and/or spelling deficits: an ERP study. Int. J. Psychophysiol. 130, 53–62. 10.1016/j.ijpsycho.2018.05.009 29803515

[B48] Korinth S. P. Breznitz Z. (2014). Fast and slow readers of the Hebrew language show divergence in brain response ~200 ms post stimulus: an ERP study. PLoS ONE 9, e103139. 10.1371/journal.pone.0103139 25078405 PMC4117504

[B49] Korinth S. P. Sommer W. Breznitz Z. (2012). Does silent reading speed in normal adult readers depend on early visual processes? Evidence from event-related brain potentials. Brain Lang. 120, 15–26. 10.1016/j.bandl.2011.08.003 21903250

[B50] Leppänen P. H. Lyytinen H. (1997). Auditory event-related potentials in the study of developmental language-related disorders. Audiol. Neurootol. 2, 308–340. 10.1159/000259254 9390838

[B51] Li S. Lee K. Zhao J. Yang Z. He S. Weng X. (2013). Neural competition as a developmental process: early hemispheric specialization for word processing delays specialization for face processing. Neuropsychologia 51, 950–959. 10.1016/j.neuropsychologia.2013.02.006 23462239 PMC3756286

[B52] Lin S. E. Chen H. C. Zhao J. Li S. He S. Weng X. C. (2011). Left-lateralized N170 response to unpronounceable pseudo but not false Chinese characters—the key role of orthography. Neuroscience 190, 200–206. 10.1016/j.neuroscience.2011.05.071 21704128

[B53] Luck S. J. (2012). Event-related potentials, in APA Handbook of Research Methods in Psychology, Vol 1: Foundations, Planning, Measures, and Psychometrics APA Handbooks in Psychology®, eds Cooper H. Camic P. M. Long D. L. Panter A. T. Rindskopf D. Sher K. J. (Washington, DC: American Psychological Association), 523–546. 10.1037/13619-028

[B54] Mahé G. Bonnefond A. Doignon-Camus N. (2013). Is the impaired N170 print tuning specific to developmental dyslexia? A matched reading-level study with poor readers and dyslexics. Brain Lang. 127, 539–544. 10.1016/j.bandl.2013.09.012 24148146

[B55] Mahé G. Bonnefond A. Gavens N. Dufour A. Doignon-Camus N. (2012). Impaired visual expertise for print in French adults with dyslexia as shown by N170 tuning. Neuropsychologia 50, 3200–3206. 10.1016/j.neuropsychologia.2012.10.013 23088819

[B56] Maurer U. Blau V. C. Yoncheva Y. N. McCandliss B. D. (2010). Development of visual expertise for reading: rapid emergence of visual familiarity for an artificial script. Dev. Neuropsychol. 35, 404–422. 10.1080/87565641.2010.480916 20614357 PMC3008655

[B57] Maurer U. Brem S. Bucher K. Brandeis D. (2005). Emerging neurophysiological specialization for letter strings. J. Cogn. Neurosci. 17, 1532–1552. 10.1162/089892905774597218 16269095

[B58] Maurer U. Brem S. Bucher K. Kranz F. Benz R. Steinhausen H.-C. . (2007). Impaired tuning of a fast occipito-temporal response for print in dyslexic children learning to read. Brain 130, 3200–3210. 10.1093/brain/awm193 17728359

[B59] Maurer U. Brem S. Kranz F. Bucher K. Benz R. Halder P. . (2006). Coarse neural tuning for print peaks when children learn to *read*. 10, 749–758. 10.1016/j.neuroimage.2006.06.025 16920367

[B60] Maurer U. McCandliss B. D. (2008). The development of visual expertise for words: the contribution of electrophysiology, in Single-Word Reading: Behavioral and Biological Perspectives, eds Grigorenko E. L. Naples A. J. (New York, NY: Lawrence Erlbaum Associates Publishers), 43–63.

[B61] Maurer U. Rossion B. McCandliss B. D. (2008a). Category specificity in early perception: face and word N170 responses differ in both lateralization and habituation properties. Front. Hum. Neurosci. 2, 18. 10.3389/neuro.09.018.2008 19129939 PMC2614860

[B62] Maurer U. Schulz E. Brem S. der Mark S. van Bucher K. Martin E. . (2011). The development of print tuning in children with dyslexia: evidence from longitudinal ERP data supported by fMRI. Neuroimage 57, 714–722. 10.1016/j.neuroimage.2010.10.055 21040695

[B63] Maurer U. Zevin J. D. McCandliss B. D. (2008b). Left-lateralized N170 effects of visual expertise in reading: evidence from Japanese syllabic and logographic scripts. J. Cogn. Neurosci. 20, 1878–1891. 10.1162/jocn.2008.20125 18370600 PMC4416222

[B64] McCandliss B. D. Cohen L. Dehaene S. (2003). The visual word form area: expertise for reading in the fusiform gyrus. Trends Cogn. Sci. 7, 293–299. 10.1016/S1364-6613(03)00134-7 12860187

[B65] Mercure E. Ashwin E. Dick F. Halit H. Auyeung B. Baron-Cohen S. . (2009). IQ, fetal testosterone and individual variability in children's functional lateralization. Neuropsychologia 47, 2537–2543. 10.1016/j.neuropsychologia.2009.04.027 19422841

[B66] Mercure E. Kadosh K. C. Johnson M. H. (2011). The N170 shows differential repetition effects for faces, objects, and orthographic stimuli. Front. Hum. Neurosci. 5, 6. 10.3389/fnhum.2011.00006 21283529 PMC3031024

[B67] Modesti P. A. Reboldi G. Cappuccio F. P. Agyemang C. Remuzzi G. Rapi S. . (2016). Panethnic differences in blood pressure in europe: a systematic review and meta-analysis. PLoS ONE 11, e0147601. 10.1371/journal.pone.0147601 26808317 PMC4725677

[B68] Moher D. Liberati A. Tetzlaff J. Altman D. G. Group T. P. (2009). Preferred reporting items for systematic reviews and meta-analyses: the PRISMA statement. PLoS Med. 6, e1000097. 10.1371/journal.pmed.1000097 19621072 PMC2707599

[B69] Niileksela C. R. Templin J. (2019). Identifying dyslexia with confirmatory latent profile analysis. Psychol. Schools 56, 335–359. 10.1002/pits.22183

[B70] Okumura Y. Kasai T. Murohashi H. (2014). Early print-tuned ERP response with minimal involvement of linguistic processing in Japanese Hiragana strings. Neuro Rep. 25, 410–414. 10.1097/WNR.0000000000000110 24356106

[B71] Okumura Y. Kasai T. Murohashi H. (2015). Attention that covers letters is necessary for the left-lateralization of an early print-tuned ERP in Japanese hiragana. Neuropsychologia 69, 22–30. 10.1016/j.neuropsychologia.2015.01.026 25613647

[B72] Ouzzani M. Hammady H. Fedorowicz Z. Elmagarmid A. (2016). Rayyan—a web and mobile app for systematic reviews. Syst. Rev. 5, 210. 10.1186/s13643-016-0384-4 27919275 PMC5139140

[B73] Page M. J. McKenzie J. E. Bossuyt P. M. Boutron I. Hoffmann T. C. Mulrow C. D. . (2021). The prisma 2020 statement: an updated guideline for reporting systematic reviews. BMJ 372, 71. 10.31222/osf.io/v7gm2 33782057 PMC8005924

[B74] Paul M. Govaart G. H. Schettino A. (2021). Making ERP research more transparent: guidelines for preregistration. Int. J. Psychophysiol. 164, 52–63. 10.1016/j.ijpsycho.2021.02.016 33676957

[B75] Pegado F. Comerlato E. Ventura F. Jobert A. Nakamura K. Buiatti M. . (2014). Timing the impact of literacy on visual processing. Proc. Natl. Acad. Sci. U. S. A. 111, E5233–E5242. 10.1073/pnas.1417347111 25422460 PMC4267394

[B76] Picton T. W. Bentin S. Berg P. Donchin E. Hillyard S. A. Johnson R. . (2000). Guidelines for using human event-related potentials to study cognition: recording standards and publication criteria. Psychophysiology 37, 127–152. 10.1111/1469-8986.3720127 10731765

[B77] Pleisch G. Karipidis I. I. Brem A. Röthlisberger M. Roth A. Brandeis D. . (2019). Simultaneous EEG and fMRI reveals stronger sensitivity to orthographic strings in the left occipito-temporal cortex of typical versus poor beginning readers. Dev. Cogn. Neurosci. 40, 100717. 10.1016/j.dcn.2019.100717 31704655 PMC6974919

[B78] Price C. J. Moore C. Humphreys G. W. Wise R. J. S. (1997). Segregating semantic from phonological processes during reading. J. Cogn. Neurosci. 9, 727–733. 10.1162/jocn.1997.9.6.727 23964595

[B79] Qin R. Maurits N. Maassen B. (2016). N170 tuning in Chinese: logographic characters and phonetic Pinyin script. Sci. Stud. Read. 20, 363–374. 10.1080/10888438.2016.1199554

[B80] Rossion B. Joyce C. A. Cottrell G. W. Tarr M. J. (2003). Early lateralization and orientation tuning for face, word, and object processing in the visual cortex. Neuroimage 20, 1609–1624. 10.1016/j.neuroimage.2003.07.010 14642472

[B81] Rumsey J. M. Horwitz B. Donohue B. C. Nace K. Maisog J. M. Andreason P. (1997). Phonological and orthographic components of word recognition. A PET-rCBF study. Brain 120, 739–759. 10.1093/brain/120.5.739 9183247

[B82] Sacchi E. Laszlo S. (2016). An event-related potential study of the relationship between N170 lateralization and phonological awareness in developing readers. Neuropsychologia 91, 415–425. 10.1016/j.neuropsychologia.2016.09.001 27614290

[B83] Savill N. J. Thierry G. (2011a). Reading for sound with dyslexia: evidence for early orthographic and late phonological integration deficits. Brain Res. 1385, 192–205. 10.1016/j.brainres.2011.02.012 21316349

[B84] Savill N. J. Thierry G. (2011b). Electrophysiological evidence for impaired attentional engagement with phonologically acceptable misspellings in developmental dyslexia. Front. Psychol. 2, 139. 10.3389/fpsyg.2011.00139 21734903 PMC3124829

[B85] Schmalz X. Marinus E. Coltheart M. Castles A. (2015). Getting to the bottom of orthographic depth. Psychon. Bull. Rev. 22, 1614–1629. 10.3758/s13423-015-0835-2 25893713

[B86] Seymour P. H. K. Aro M. Erskine J. M. (2003). Foundation literacy acquisition in European orthographies. Br. J. Psychol. 94, 143–174. 10.1348/000712603321661859 12803812

[B87] Shaywitz B. A. Shaywitz S. E. Pugh K. R. Mencl W. E. Fulbright R. K. Skudlarski P. . (2002). Disruption of posterior brain systems for reading in children with developmental dyslexia. Biol. Psychiatry 52, 101–110. 10.1016/S0006-3223(02)01365-3 12114001

[B88] Shaywitz S. E. Shaywitz B. A. Pugh K. R. Fulbright R. K. Constable R. T. Mencl W. E. . (1998). Functional disruption in the organization of the brain for reading in dyslexia. Proc. Natl. Acad. Sci. U. S. A. 95, 2636–2641. 10.1073/pnas.95.5.2636 9482939 PMC19444

[B89] Simon G. Petit L. Bernard C. Rebaï M. (2007). N170 ERPs could represent a logographic processing strategy in visual word recognition. Behav. Brain Funct. 3, 21. 10.1186/1744-9081-3-21 17451598 PMC1884163

[B90] Snowling M. J. (2013). Early identification and interventions for dyslexia: a contemporary view. J. Res. Spec. Educ. Needs 13, 7–14. 10.1111/j.1471-3802.2012.01262.x 26290655 PMC4538781

[B91] Spironelli C. Angrilli A. (2009). Developmental aspects of automatic word processing: language lateralization of early ERP components in children, young adults and middle-aged subjects. Biol. Psychol. 80, 35–45. 10.1016/j.biopsycho.2008.01.012 18343558

[B92] Spironelli C. Penolazzi B. Angrilli A. (2010). Gender differences in reading in school-aged children: an early ERP study. Dev. Neuropsychol. 35, 357–375. 10.1080/87565641.2010.480913 20614355

[B93] Strijkers K. Yum Y. N. Grainger J. Holcomb P. J. (2011). Early Goal-directed top-down influences in the production of speech. Front. Psychol. 2, 371. 10.3389/fpsyg.2011.00371 22163224 PMC3234706

[B94] Su M. Wang J. Maurer U. Zhang Y. Li J. McBride C. . (2015). Gene–environment interaction on neural mechanisms of orthographic processing in Chinese children. J. Neurolinguistics 33, 172–186. 10.1016/j.jneuroling.2014.09.007 26294811 PMC4539967

[B95] Taha H. Ibrahim R. Khateb A. (2013). How does Arabic orthographic connectivity modulate brain activity during visual word recognition: an ERP study. Brain Topogr. 26, 292–302. 10.1007/s10548-012-0241-2 22864655

[B96] Taha H. Khateb A. (2013). Resolving the orthographic ambiguity during visual word recognition in Arabic: an event-related potential investigation. Front. Hum. Neurosci. 7, 821. 10.3389/fnhum.2013.00821 24348367 PMC3845210

[B97] Tong X. Lo J. C. M. McBride C. Ho C. S. Waye M. M. Y. Chung K. K. H. . (2016a). Coarse and fine N1 tuning for print in younger and older Chinese children: orthography, phonology, or semantics driven? Neuropsychologia 91, 109–119. 10.1016/j.neuropsychologia.2016.08.006 27507119

[B98] Tong X. Maurer U. Chung K. K. H. McBride C. (2016b). Neural specialization for print in Chinese-English language learners. J. Neurolinguistics 38, 42–55. 10.1016/j.jneuroling.2015.10.001

[B99] Torppa M. Tolvanen A. Poikkeus A.-M. Eklund K. Lerkkanen M.-K. Leskinen E. . (2007). Reading development subtypes and their early characteristics. Ann. Dyslexia 57, 3–32. 10.1007/s11881-007-0003-0 17849214

[B100] Uno T. Okumura Y. Kasai T. (2017). Print-specific N170 involves multiple subcomponents for Japanese Hiragana. Neurosci. Lett. 650, 77–81. 10.1016/j.neulet.2017.04.020 28412533

[B101] van Setten E. R. H. Martinez-Ferreiro S. Maurits N. M. Maassen B. A. M. (2016). Print-tuning lateralization and handedness: an event-related potential Study in dyslexic higher education students. Dyslexia 22, 64–82. 10.1002/dys.1519 26639313

[B102] van Setten E. R. H. Maurits N. M. Maassen B. A. M. (2019). N1 lateralization and dyslexia: an event-related potential study in children with a familial risk of dyslexia. Dyslexia 25, 84–102. 10.1002/dys.1604 30407716 PMC6587992

[B103] Van Strien J. W. Glimmerveen J. C. Martens V. E. G. De Bruin E. A. (2009). Age-related differences in brain activity during extended continuous word recognition in children. Neuroimage 47, 688–699. 10.1016/j.neuroimage.2009.05.020 19446639

[B104] Waldie K. E. Badzakova-Trajkov G. Lim V. K. Kirk I. J. (2012). Lexical decision making in adults with dyslexia: an event-related potential study. Ilha Desterro J. Engl. Lang. Lit. Engl. Cult. Stud. 63, 37–68. 10.5007/2175-8026.2012n63p37

[B105] Wei D. Gillon Dowens M. Guo T. (2018). Early lexical processing of Chinese words indexed by Visual Mismatch Negativity effects. Sci. Rep. 8, 1289. 10.1038/s41598-018-19394-y 29358675 PMC5778037

[B106] Wolff U. (2010). Subgrouping of readers based on performance measures: a latent profile analysis. Read. Writ. 23, 209–238. 10.1007/s11145-008-9160-8

[B107] Wong A. C. N. Gauthier I. Woroch B. Debuse C. Curran T. (2005). An early electrophysiological response associated with expertise in letter perception. Cogn. Affect. Behav. Neurosci. 5, 306–318. 10.3758/CABN.5.3.306 16396092

[B108] Xue L. Maurer U. Weng X. Zhao J. (2019). Familiarity with visual forms contributes to a left-lateralized and increased N170 response for Chinese characters. Neuropsychologia 134, 107194. 10.1016/j.neuropsychologia.2019.107194 31542360

[B109] Yang H. Zhao J. Gaspar C. M. Chen W. Tan Y. Weng X. (2017). Selectivity of N170 for visual words in the right hemisphere: evidence from single-trial analysis. Psychophysiology 54, 1128–1137. 10.1111/psyp.12867 28369927

[B110] Yum Y. N. Holcomb P. J. Grainger J. (2011). Words and pictures: an electrophysiological investigation of domain specific processing in native Chinese and English speakers. Neuropsychologia 49, 1910–1922. 10.1016/j.neuropsychologia.2011.03.018 21439991 PMC3100363

[B111] Zhao J. Kipp K. Gaspar C. Maurer U. Weng X. Mecklinger A. . (2014). Fine neural tuning for orthographic properties of words emerges early in children reading alphabetic script. J. Cogn. Neurosci. 26, 2431–2442. 10.1162/jocn_a_00660 24800627

[B112] Zhao J. Maurer U. He S. Weng X. (2019). Development of neural specialization for print: evidence for predictive coding in visual word recognition. PLoS Biol. 17, e3000474. 10.1371/journal.pbio.3000474 31600192 PMC6805000

[B113] Zhao P. Li S. Zhao J. Gaspar C. M. Weng X. (2015). Training by visual identification and writing leads to different visual word expertise N170 effects in preliterate Chinese children. Dev. Cogn. Neurosci. 15, 106–116. 10.1016/j.dcn.2015.09.002 26409757 PMC6989826

[B114] Zhao P. Zhao J. Weng X. Li S. (2018). Event-related potential evidence in Chinese children: type of literacy training modulates neural orthographic sensitivity. Int. J. Behav. Dev. 42, 311–320. 10.1177/0165025417708341

[B115] Zhou X. Ye Z. Cheung H. Chen H.-C. (2009). Processing the Chinese language: an introduction. Lang. Cogn. Process. 24, 929–946. 10.1080/01690960903201281

